# Health effects of particulate matter air pollution in underground railway systems – a critical review of the evidence

**DOI:** 10.1186/s12989-019-0296-2

**Published:** 2019-03-06

**Authors:** Matthew Loxham, Mark J. Nieuwenhuijsen

**Affiliations:** 1 Academic Unit of Clinical and Experimental Sciences, Faculty of Medicine, University of Southampton, Mailpoint 888, Level F, University Hospital Southampton, Tremona Road, Southampton, SO16 6YD UK; 2 NIHR Southampton Biomedical Research Centre, University Hospital Southampton, Southampton, UK; 3 Institute for Life Sciences, University of Southampton, Southampton, UK; 4 Southampton Marine and Maritime Institute, University of Southampton, Southampton, UK; 5 ISGlobal, Centre for Research in Environmental Epidemiology (CREAL), Barcelona, Spain; 6 Universitat Pompeu Fabra (UPF), Barcelona, Spain; 7 CIBER Epidemiología y Salud Pública (CIBERESP), Madrid, Spain

**Keywords:** Underground railway, Subway, Particulate matter, Transition metal, Steel, Iron, Reactive oxygen species, Oxidative stress, Inflammation

## Abstract

**Background:**

Exposure to ambient airborne particulate matter is a major risk factor for mortality and morbidity, associated with asthma, lung cancer, heart disease, myocardial infarction, and stroke, and more recently type 2 diabetes, dementia and loss of cognitive function. Less is understood about differential effects of particulate matter from different sources. Underground railways are used by millions of people on a daily basis in many cities. Poor air exchange with the outside environment means that underground railways often have an unusually high concentration of airborne particulate matter, while a high degree of railway-associated mechanical activity produces particulate matter which is physicochemically highly distinct from ambient particulate matter. The implications of this for the health of exposed commuters and employees is unclear.

**Main body:**

A literature search found 27 publications directly assessing the potential health effects of underground particulate matter, including in vivo exposure studies, in vitro toxicology studies, and studies of particulate matter which might be similar to that found in underground railways. The methodology, findings, and conclusions of these studies were reviewed in depth, along with further publications directly relevant to the initial search results.

In vitro studies suggest that underground particulate matter may be more toxic than exposure to ambient/urban particulate matter, especially in terms of endpoints related to reactive oxygen species generation and oxidative stress. This appears to be predominantly a result of the metal-rich nature of underground particulate matter, which is suggestive of increased health risks. However, while there are measureable effects on a variety of endpoints following exposure in vivo, there is a lack of evidence for these effects being clinically significant as may be implied by the in vitro evidence.

**Conclusion:**

There is little direct evidence that underground railway particulate matter exposure is more harmful than ambient particulate matter exposure. This may be due to disparities between in vivo exposures and in vitro models, and differences in exposure doses, as well as statistical under powering of in vivo studies of chronic exposure. Future research should focus on outcomes of chronic in vivo exposure, as well as further work to understand mechanisms and potential biomarkers of exposure.

## Background

Underground railways are mass transit systems used for several million passenger journeys per day in many of the world’s most populous cities [1]. Depending on station depth, proximity to the nearest transition from tunnel section to the outside environment, and air conditioning system, there may be relatively little exchange of air with the outside environment, and this may be further modulated by station design [2–7]. As such, the airborne particulate matter (PM) load in underground railway stations has the potential to be (1) significantly greater, on a mass concentration basis, than the outside environment, and (2) influenced principally by source materials and generation methods specific to the underground railway. These processes include wear of wheels and brakes, and arcing of electrical current between the third rail or catenary and the current collecting apparatus [8, 9]. As a result, PM generated in underground railway systems tends to be rich in metals, especially Fe, but also Cr, Ni, Co, Mn, and Cd, across coarse (PM_10–2.5_; median aerodynamic diameter 10–2.5 μm), fine (PM_2.5_; < 2.5 μm), and ultrafine (UFPM/PM_0.1_; < 0.1 μm) fractions [8, 10–12]. Given the potential for underground PM to differ significantly in composition, as well as in physical characteristics such as particle number concentration (PNC), and the number of people potentially exposed on a regular basis through either employment in underground railway systems or regular commuting, the question of whether underground PM exerts effects on health and, if so, the mechanisms involved, warrants urgent attention.

Air quality in underground systems has been the focus of previous reviews, but these have centred around pollution characterisation and chemistry, rather than potential health effects [13, 14]. Therefore, the focus of this review is the critical examination of the evidence for potential health effects of PM in underground stations, and PM which may be similar to that found in underground stations. The review is divided into four principal sections, respectively focusing on evidence for effects of exposure to underground PM in vivo, evidence from in vitro studies, studies which use various derived risk factors to evaluate in vivo risk, and evidence from in vivo studies of exposure to PM not from underground railways, but which may approximate the physicochemical characteristics of underground PM.

## Main text

### Search strategy and review structure

**Fig. 1:**
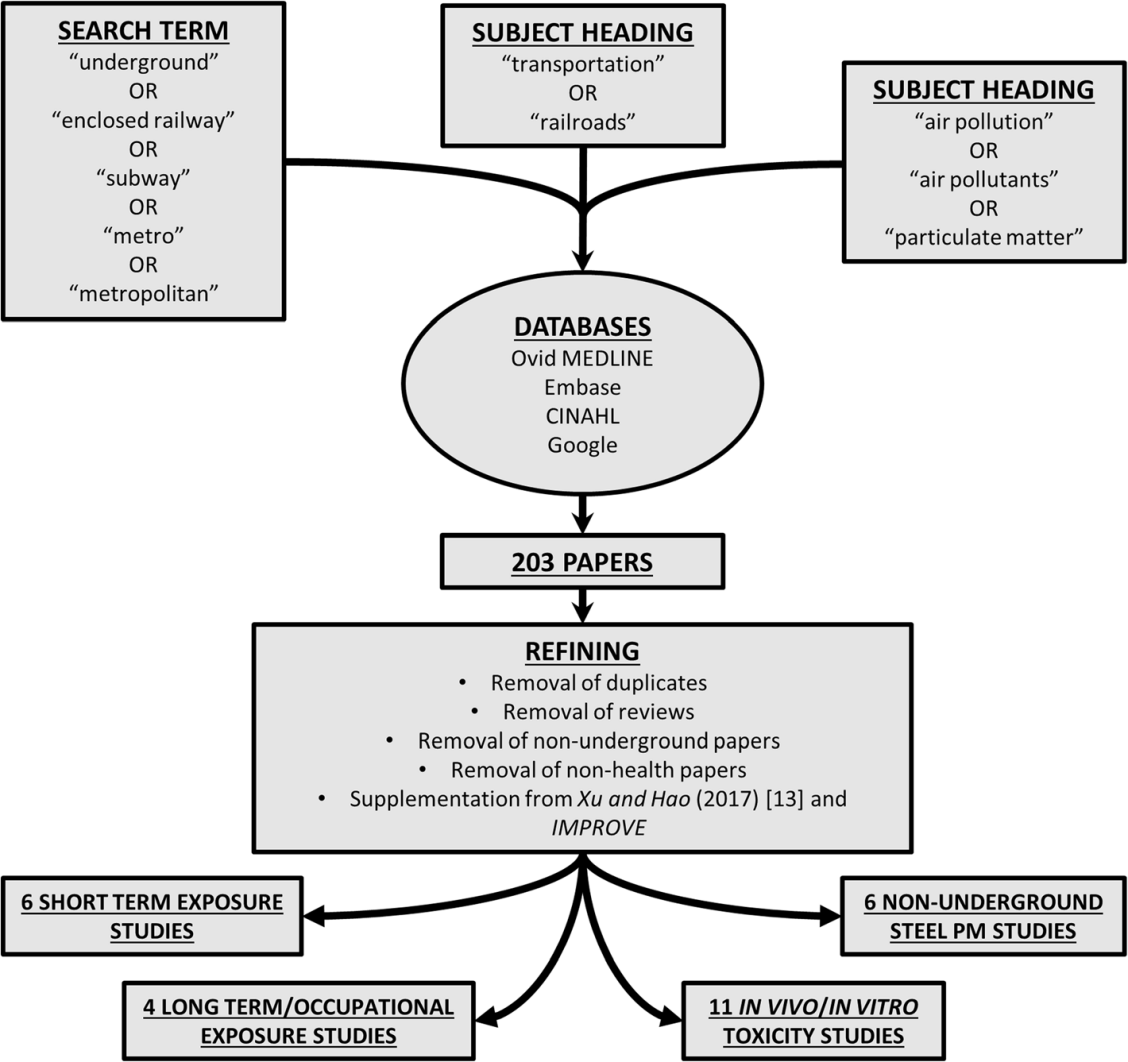
Literature search methodology. Papers were selected for inclusion in the review by combining each of the search boxes in the figure with the Boolean operator “AND”, across four databases. The 203 papers yielded by this initial search were refined to include only those of relevance to underground railways and health, and supplemented by relevant papers used in the review of Xu and Hao [13] and also the IMPROVE study. This yielded the 27 papers which form the core body of literature for this review. Further studies have been included and cited where appropriate

The search strategy for this review is shown in Fig. 1. An initial literature search was performed by Dr. Sarah Robertson (Public Health England, UK) forming the basis for a report by a subcommittee of the UK Committee on the Medical Effects of Air Pollution (COMEAP). The literature search was performed across Ovid MEDLINE, Embase, CINAHL, and Google databases, to include all published, Epub ahead of print, in-process, and non-indexed citations. The search was designed to find papers on all aspects of underground railway air quality, not limited to those studying the health effects, but also those relevant to pollution chemistry and engineering. The search was carried out for all entries containing (1) EITHER “underground” OR “enclosed railway” OR “subway” OR “metro” OR “metropolitan”, AND (2) the subject heading “transportation” OR the subject heading “railroads”, AND (3) the subject heading “air pollution” OR the subject heading “air pollutants” OR the subject heading “particulate matter”. Results containing the term “coal” were excluded. This yielded 203 results. Initial refining of results to remove duplicates and those not relevant to the underground left 51 exposure studies, 8 in vivo/in vitro toxicity studies, and 14 health studies. These results were supplemented using references from a previous review by Xu and Hao [13] and the EU-Life funded project IMPROVE. Further refining to remove reviews and those which did not have a direct health component, and rearranging into the categories used in this review, yielded 6 short term exposure studies on volunteers, 4 long term/occupational exposure studies, 11 in vivo/in vitro toxicity studies, and 6 studies focusing on steel mill or other steel-related PM. These studies form the basis of this review, with additional references selected only where specifically addressing in vitro or in vivo effects of exposure, or directly linked to/relevant to such studies.

Throughout this review, the terms “underground” and “underground railway” are used for consistency. This is equivalent to terms such as “subway” and “metro” used elsewhere, with the key distinction required for the use of the term “underground” being that the station/section in question is subterranean, unless explicitly stated.

### (1) In vivo evidence for health effects of exposure to underground air pollution/PM

#### Acute exposure

Klepczynska Nystrom et al exposed 20 healthy volunteers to a Stockholm underground environment or a control environment for 2 h and investigated effects of this exposure on lung function and inflammation in the lower airways and blood. Lung function tests found no effect of underground exposure on vital capacity (VC), forced vital capacity (FVC), forced expiratory volume in 1 s (FEV_1_), exhaled NO, or peak expiratory flow (PEF). They found statistically significantly increased levels of fibrinogen in plasma of those exposed in the underground, with increased counts of CD4/CD25/FOXP3 T cells [15]. The increase in CD4 T cells positive for FOXP3, or FOXP3 and CD25 (in blood but not bronchoalveolar lavage fluid (BALF)) indicates an increase in the pool of regulatory T cells (Treg) at 14 h following underground exposure. A key function of Treg cells is to reduce the inflammatory response, which can be achieved through secretion of anti-inflammatory cytokines and effects on other immune cells [16, 17]. As such, the increase in Treg numbers may be a response to the systemic inflammation indicated by increased fibrinogen release. Concentration of the anti-inflammatory cytokine IL-10, released by Tregs, was assayed only in the BALF (where there was no increase in Tregs) and not in the blood (where Tregs were increased). Similar increases in systemic Tregs but not lung Tregs have been found following PM_2.5_ exposure in mice [18]. Post-exposure blood tests also showed slight but significant increases in other markers of T cell activation. This study did not consider the ability of Tregs to suppress inflammation, which has been seen to be impaired by ambient air pollution exposure in children, especially those with asthma [19]. Therefore, while the increase in peripheral blood Treg cells in this study may represent a natural consequence of the systemic inflammation of which increased fibrinogen concentrations are a marker, it is impossible to determine whether there is any effect of underground railway pollution on Treg functioning.

The lack of effect on lung function parameters was also found in a study by Bigert et al, who investigated effects of short-term exposure of employees in the Stockholm underground by measuring the before-work and after-work fraction of exhaled NO (F_E_NO), and taking regular measurements of PEF and FEV_1_ [20]. These employees were mainly non-asthmatic (74/81), and were either platform workers, train drivers, or ticket office staff, in order of decreasing PM_2.5_ exposure. The authors found no change in F_E_NO over the working day in any of the occupation/exposure groups, nor was there a significant decrease in PEV/FEV_1_ ratio over the working day, which suggests no obvious decline in airway function. The authors do, however, suggest that their study design might not have allowed a sufficient washout for the inflammatory effects of previous exposures to disappear before the before-work F_E_NO testing, or that the after-work test may have been too soon to observe the response to the day’s exposure. Nonetheless, the results are in broad agreement with other tests presented here where lung function testing is used to assess responses to short-term underground exposure.

In a follow-up to their study on the effects of underground exposure in healthy individuals [15], Klepczynska Nystrom et al observed that mild asthmatics similarly exposed had increased activated T cells in BALF (identified by the marker CD25) but no increase in Tregs in the blood (unlike their healthy counterparts in the previous study) [21]. In the context of this effect on the lungs, it is notable that asthmatic volunteers, but not the healthy volunteers from the previous study, also reported increased upper airways symptoms, whereas the healthy volunteers reported increased lower airway symptoms. This might reflect increased deposition of PM in the upper airways in asthma as a result of increased mucus production and/or reduced airway patency, with reduced penetration of PM to the lower airways and alveoli, perhaps due to increased airflow turbulence as a result of the disease. However, the overall conclusions of the authors suggest that, although there were some changes in markers indicative of systemic inflammation after 2 h exposure to underground PM (healthy group) and differences in the response of a group of mild asthmatics (albeit only according to a small number of the many parameters examined), acute exposure to underground air caused only slight acute effects, with changes in biomarkers being of uncertain consequence in terms of immune system functioning. Moreover, these two studies found no significant increase in the concentrations of any inflammatory cytokines (IL-1β, IL-6, IL-8, and TNFα) or anti-inflammatory cytokine (IL-10) in BALF, suggestive of a lack of acute inflammatory response [15, 21]. Furthermore, these studies were not designed to study the effect of persistent exposure over longer periods.

Changes in systemic markers in healthy, but not asthmatic, subjects were also seen in these studies in terms of plasma fibrinogen concentrations, which increased slightly, but nonetheless significantly, in healthy volunteers from 2.2 g/l to 2.3 g/l, but did not change in mild asthmatics, although the physiological relevance of this small increase in terms of adverse cardiovascular outcomes is unclear. Fibrinogen undergoes enzymatic cleavage by thrombin to form fibrin, which then forms the “meshwork” for clot formation [22]. Because of this increased tendency towards clot formation, fibrinogen is regarded as a risk factor for a coronary heart disease, stroke, and other vascular disorders [23]. It is notable, therefore, that this study found no difference in levels of plasminogen activator inhibitor-1 (PAI-1), which is also involved in clot formation by inhibiting the activation of an enzyme cascade involved in clot breakdown [24]. This study sampled blood 14 h after exposure, which is within the normal time frame for acute response proteins to be increased in the blood, although it is possible that slightly later timepoints may reveal increased levels of such markers not seen at 14 h. In addition to C-reactive protein and various inflammatory cytokines (e.g. IL-6), fibrinogen is a commonly assayed blood-borne marker of the effects of air pollution in humans, specifically as a marker of systemic inflammation, where it is sometimes noted to be correlated with circulating levels of C-reactive protein (reviewed by [25]). The increase in fibrinogen has been suggested to be dependent on the genotype of the individual concerned, for exposure to gaseous pollutants, although the same may not necessarily be true for PM pollution [26]. Bigert and colleagues found increased fibrinogen in underground ticket sellers and PAI-1 in underground train drivers after 2 days of work following a work-free wash-out period of at least 2 days [27]. However, baseline levels were highest in platform workers, who showed no acute increases in these markers, suggesting that acute changes were not due to PM exposure. This is in line with their findings in the same cohort used for their previous study, detailed above, which showed no change in lung function or inflammation by F_E_NO or PEV/FEV_1_ measurement [20]. Therefore, while fibrinogen is commonly used as a biomarker for systemic inflammation as a result of short term exposure to PM, the evidence for a biologically significant effect of underground PM on fibrinogen is lacking.

In contrast Lundstrom et al [28] used lipid mediators in BALF to study the effects of exposure to underground air. These are not commonly used markers in such studies, perhaps on account of the numerous analytes studied, the need for lavage, the requirement for specialised mass spectrometry techniques for their measurement, or their lack of comparability to other studies. The study showed an increased level of nine oxylipins out of sixty-four assayed in healthy volunteers compared to mild asthmatic volunteers, following 2 h exposure in the Stockholm underground. Levels of these oxylipins increased in the healthy volunteers, while tending to remain the same or slightly decrease in the mild asthmatic volunteers. These nine oxylipins were prostaglandin E2 (PGE2; a product of arachidonic acid metabolism by cyclooxygenase), and eight products of biosynthesis from linoleic or α-linolenic acids via 15-lipoxygenase, suggesting a common mechanism of regulation. The authors suggested that, given the bronchoprotective effects of the oxylipins increased in healthy individuals but not asthmatics following exposure to underground air, there is evidence of a differential effect depending on asthma status. This is supported by the increased BAL cell expression of cyclooxygenase-1 in healthy volunteers only. However, while these results imply asthma status-specific effects, the pathways of these metabolites are significantly complex that it is difficult to draw any robust conclusions regarding their implications for acute responses to underground air exposure. Furthermore, the effects of the observed changes in their concentration is unclear, and it is also noteworthy that changes were observed in only a small proportion of the mediators assayed. Lipid mediators and lipid oxidation in general represent an interesting and currently under-investigated avenue for PM research, but this study is not sufficient to draw any relevant conclusion other than as an illustration that some lipid mediators may change in response to underground air exposure, and that there are asthmatic vs. healthy differences in response to the underground environment. The lack of research into lipid mediators following pollution exposure is underlined in a recent paper on the effects of biodiesel exhaust [29], where healthy volunteers were exposed to biodiesel exhaust at a PM level not dissimilar to the PM level of the Stockholm underground. They found increased concentrations in BALF of PGE2, 13-hydroxyoctadecadienoic acid (13-HODE), and 12,13-dihydroxyoctadecadienoic acid (12,13-diHOME) following biodiesel exhaust exposure. Interestingly, while PGE2 and 13-HODE were significantly more responsive to underground air exposure in healthy vs. asthmatic volunteers in the Stockholm underground study and 12,13-diHOME was close to significance (*p* = 0.1), none of these were significantly increased in the Stockholm underground following exposure vs. no exposure, and further examination of the Stockholm underground study shows that the significance threshold for analysis between asthma status was likely attained not simply because of an increase in levels of these oxylipins in healthy lavage fluid, but also because of a decrease in their levels in asthmatics. Indeed, considering the asthmatic and healthy groups separately, only two oxylipins were significantly altered by exposure to underground air (one of these overlapped both groups). This further reinforces the conclusion that the Stockholm study, while suggesting some disease-related differences, does not provide strong evidence per se for any significant effect of underground air, but rather evidence of some more subtle asthmatic vs. non-asthmatic differences.

A study by Liu et al used heart rate variability (HRV) as an endpoint [30]. HRV is often used as an endpoint to assess the effects of pollution, representing a risk factor, both in itself and as a marker of autonomic imbalance, for a number of adverse cardiovascular outcomes [31], and is thought to be influenced by effects of pollution on the autonomic nervous system, resulting in disturbed sympathetic tone, and thus altered sympathetic/parasympathetic balance. This study found a decrease in HRV with increasing PM_2.5_ exposure, as has been noted elsewhere (for a comprehensive overview, see recent review by Kelly and Fussell [32]). Interestingly, the study noted that the decrease in HRV seen by participants using the underground for a 1 h commute was less than seen for those walking or using the car or bus. However, the concentrations of PM_2.5_ to which participants were exposed shows that the underground air was significantly less polluted than environments used for walking or bus exposures, and trended to a lower concentration compared to car-based exposures. This was also true for exposure to PM_10_ and total volatile organic compounds. The finding that the underground was the least PM-loaded environment is somewhat unusual - one reason for the relatively low PM concentration noted in the Taipei underground here may be the use of an air conditioning system. Therefore, it is difficult to extrapolate these results to understanding the effects of exposures in other underground networks, where the air tends to be more PM-rich, although the study does imply that when similar PM_10_ and PM_2.5_ mass concentrations are involved, air in the underground is not necessarily more detrimental than other modes of transport, at least in terms of HRV.

#### Chronic exposure

There is a paucity of studies of the effects of chronic exposure to underground railway pollution. Bigert and colleagues found no significant increased risk of myocardial infarction in 304 underground train drivers amongst a study population of 153,807 men aged 40–69 in Stockholm County [33]. Similarly, Gustavsson and colleagues found that the incidence of lung cancer was not increased in 348 Stockholm underground drivers compared to 319,979 employed males in the Stockholm area [34]. However, these studies may have been insufficiently powered to uncover an effect, on account of the relatively small number of underground train drivers and low incidence of disease (54 myocardial infarction cases and 9 lung cancer cases in the two studies, respectively). Indeed, both studies have somewhat large confidence intervals of relative risk. In a pilot study of workers on the New York underground, Grass and colleagues found that track maintenance and construction workers were exposed to higher concentrations of PM_2.5_ and airborne Fe, Mn, and Cr compared to other underground workers, although drivers and train flaggers were exposed to the highest concentrations of Fe when expressed as a proportion of PM exposure [35]. Using bus drivers and office workers as control groups, they found little evidence of adverse health effects of working underground using a range of urinary and plasma biomarkers, predominantly metal- and oxidative stress-related. Urinary Mn and 8-hydroxy-2′-deoxyguanosine (8-OHdG, a marker of oxidative DNA damage) concentrations were highest in office workers, while urinary concentrations of the polyaromatic hydrocarbon (PAH) metabolite benzo[a]pyrene diol epoxide (BDPE) were highest in bus drivers. Urinary 8-isoprostane levels were non-significantly higher in underground workers than the control groups, although in the underground group there was a significant correlation with total years of underground work, suggesting a potential effect of cumulative exposure. Plasma concentrations of protein carbonyls (a marker of oxidative damage to proteins) were no different between underground workers and bus drivers, and lower for office workers, although they were correlated with plasma Mn in subway drivers but not the control groups, possibly indicating different causes of protein oxidation below and above ground. Plasma Mn was similar across all groups, while plasma concentrations of Cr and DNA-protein crosslinks were lower in bus drivers but not office workers, and there was no difference in plasma Pb. As such, this study showed no obvious effect of underground exposures to any of the urinary or plasma biomarkers measured, and nor did raised concentrations of Mn or Cr underground necessarily translate to commensurately raised levels of these elements in the plasma of exposed workers. Conversely, Mehrdad and colleagues found that there was a small but statistically significant increase in urinary concentration of the DNA oxidation biomarker 8-OHdG in underground tunnel workers compared with underground workers who did not work in the tunnels, after correction for age, BMI, disease, and smoking, although they were unable to correct for alcohol consumption, nor were they able to measure PM_2.5_ or steel dust exposure in either of the two groups [36]. Furthermore, it is not clear whether this difference represents a response to acute exposure over a shift, or accumulation of chronic exposure, since no pre-shift measurements were taken.

#### Summary

These studies are summarised in Table 1. Studies aimed at understanding the effects of exposure to underground railway air pollution have generally found no consistent, convincing evidence for significant effects on the health of either acute (2–8 h) or chronic exposure. Studies of acute exposure in the Stockholm underground found no effect on lung function, although there were some reported lower (healthy volunteers) and upper (asthmatic volunteers) airway symptoms. These studies did show effects on Treg cell populations following exposure, but the clinical significance of this, along with the observed slight increase in circulating fibrinogen, is unclear, especially in light of the lack of other observable effects. Increased concentrations of a relatively small number of oxylipins in BALF may suggest differential effects depending on asthma status, but again the clinical significance is unclear. Studies of the effects of chronic exposure have been similarly lacking in evidence, suggesting no increased risk of myocardial infarction or lung cancer, and noting no obvious effect of underground exposure on a range of circulating biomarkers which one might hypothesise to be affected, were underground exposure to be a significant cause for concern. However, it is also possible that these studies are underpowered, with only a small number of cases, and commensurately large confidence intervals in their results. Therefore, although these studies provide no clear evidence for significant health effects of acute or chronic exposure in the underground, there is clearly a need for larger studies and studies better powered to analyse differential effects on those with underlying airway and cardiovascular disease. Furthermore, there is clearly a disconnect between the results of these in vivo studies, and those which use a range of other techniques, such as in vitro models and in vitro-in vivo extrapolation, as detailed the in the sections which follow.

### (2) In vitro studies of the toxicity of underground air pollution

**Table 1:** Studies investigating the health effects of exposure to underground railway air pollution in vivo

First Author	Publication Year (Study Dates)	Underground	[Airborne PM] (μg/m^3^ unless stated)	PM Composition	Exposure Period	Sample Size	Effects of Underground Exposure
Klepczynska Nystrom [15]	2010 (October 2006–March 2007)	Stockholm, Sweden	PM_10_ = 242 ± 40; PM_2.5_ = 77 ± 10; PM_0.1_ PNC = 8283 ± 1716/cm^3^	PM_10_: Fe = 58.6 ± 21.0%; Ba = 1.0 ± 0.4%; Cu = 0.8 ± 0.4%; Mn = 0.5 ± 0.2%	2 h, afternoon rush hour	20 healthy non-smoking volunteers (13 M 7F), mean age 27 y (range 18–46)	No change in lung function or airway cellular parameters, increased plasma fibrinogen, increased blood Treg count.
Bigert [20]	2011 (November 2004–March 2005)	Stockholm, Sweden	PM_10–1_ DataRAM and PM_2.5_ ticket office = 13 ± 3, 10 ± 3; train drivers = 33 ± 12, 19 ± 3; platform workers = 182 ± 57, 63 ± 12	Not stated	~ 8 h working day	81 non-smoking workers (55 M 26F), mean age 38 y (range 25–50)	No changes in F_E_NO or lung function over working day.
Klepczynska Nystrom [21]	2012 (mid November-early April, year not stated)	Stockholm, Sweden	PM_10_ = 232 ± 51; PM_2.5_ = 71 ± 13; PM_0.1_ PNC = 8960 ± 660/cm^3^	PM_10_: Fe = 49.3 ± 7.3%; Ba0.7 ± 0.1%; Cu = ND; Mn = 0.4 ± 0.1	2 h, afternoon rush hour	16 mild asthmatic non-smoking volunteers (5 M 11F), mean age 26 y (range 18–52)	Increased activated T cells in BALF, no effect on blood T cell counts or coagulation markers.
Bigert [27]	2008 (November 2004–March 2005)	Stockholm, Sweden	PM_10–1_ DataRAM and PM2.5 ticket sellers = 13 ± 3, 10 ± 3; train drivers = 33 ± 12, 19 ± 3; platform cleaners = 256 ± 97, 79 ± 17; platform ticket collectors = 108 ± 26, 50 ± 8	Not stated	48 h (over 2 working days)	79 non-smoking workers (54 M, 25F), mean age 38 y (range 25–50)	Increased PAI-1 in ticket sellers, increased fibrinogen in train drivers. More exposed platform workers had higher baseline PAI-1 and hsCRP, but no effect over exposure period. No obvious PM effect.
Lundstrom [28]	2011 (mid November-early April, year not stated)	Stockholm, Sweden	Not stated – see [15, 21]	Not stated – see [15, 21]	2 h, afternoon rush hour	18 healthy, 15 mild intermittent asthmatic non-smoking volunteers (17 M, 16F), mean age 26 y (range 18–52)	9/64 oxylipins assayed in BALF increased in healthy vs. asthmatic, volunteers, principally 15-lipoxygenase-generated derivatives of linoleic and α-linolenic acids.
Liu [30]	2015	Taipei, Taiwan	PM_10_, PM_2.5_ underground = 32 ± 12, 22 ± 7; bus = 40 ± 16, 32 ± 12; car = 34 ± 13, 29 ± 11; walking = 50 ± 21, 42 ± 18	Not stated	1 h morning commute	120 healthy volunteers (58 M, 62F), mean age 21 y (range 19–24)	Underground commute showed lowest PM_2.5_ exposure and lowest effect on heart rate variability vs. bus, car, or walk.
Bigert [33]	2007 (data from 1976 to 1996)	Stockholm, Sweden	Not stated	Not stated	Chronic workplace exposure	131,496 M (250 underground drivers), 22,311 myocardial infarction cases (54 underground drivers)	No increased risk of myocardial infarction in underground drivers (RR = 0.92 [95% CI 0.68–1.25] vs. manual workers, 1.06 [95% CI 0.78–1.43] vs. other employed males).
Gustavsson [34]	2008 (subjects followed from 1970 to 1989)	Stockholm, Sweden	Not stated	Not stated	Chronic workplace exposure	319,979 M (348 underground drivers), 4731 lung cancer cases (9 underground drivers)	No increased risk of lung cancer in underground drivers (standardised incidence ratio 0.82 [95% CI 0.38–1.56]).
Grass [35]	2010 (November 2004–February 2005)	New York City, USA	PM_2.5_ exposure median across all subway roles = 27 (5th–95th %ile = 8–112)	PM_2.5_: median Fe = 27%	Chronic workplace exposure	39 M underground drivers (median age 48 y, IQR 38–53), 11 M bus drivers (45 y, 41–48), 25 M office workers (44 (37–51))	Across a wide range of chemical and biomarker assays in blood and urine, only urinary 8-isoprostate was associated with (cumulative) underground exposure.
Mehrdad [36]	2015 (September–October 2012)	Tehran, Iran	Not stated	Not stated	Chronic workplace exposure	81 M healthy underground workers, mean age 32 ± 7 y	Increased urinary 8-OHdG in underground tunnel workers vs. underground non-tunnel workers.

Several non-human studies (mainly in vitro) have been conducted to examine the toxicity of underground PM and mechanisms involved. While they cannot give the same insight into potential effects on exposed humans as those studies in the previous section, they may be better able to shed light on the potential cellular mechanisms at work, and how they relate to the composition of the PM, which is important in understanding whether underground PM may exert effects different to those caused by urban PM, and why [37].

Seaton et al examined the characteristics of PM on the London Underground from a size and compositional point of view, with PM collection and monitoring at three deep-level underground stations (Oxford Circus, Holland Park, Hampstead) [38]. The mass concentration of PM_2.5_ underground was noted to be much higher than would be seen above ground, but there was not a correspondingly higher level of PM by PNC. In fact, the number concentration was lower than might be expected in a city street. Overall, the size distribution of underground PM vs. urban PM was shifted towards the larger size of the PM spectrum. If composition is disregarded and only size/PNC is considered, this observation suggests that underground PM may well pose less of a health risk that that above ground, where higher numbers of particulates persist, given that PNC has been suggested to be more important a dose metric than particle mass [39]. Furthermore, the transition metal-rich nature of underground PM means that it is likely denser than urban PM, and thus would have a lower PNC, even if the size distribution and mass concentrations were identical. However, Seaton and colleague’s work also showed that, in vitro, A549 cells (a type II alveolar epithelial carcinoma cell line) were more affected by underground PM than ambient PM or control particles (TiO_2_) in terms of release of the neutrophil chemoattractant/activating cytokine IL-8 and extent of DNA damage, as determined by plasmid scission assay. The increase in IL-8 release could be abrogated by chelation (the exact method is not stated) implying that PM surface or soluble transition metals are responsible for this effect. Furthermore, the use of welding dust as a comparator in this study was not ideal given the fact that much of the underground PM is generated by abrasion rather than high temperature processes generating fume.

Three papers by Karlsson and colleagues detail experiments similar to those of Seaton et al, using PM from the Stockholm underground, but with additional analyses [40–42]. Data from their first paper in 2005 showed that Stockholm underground PM was more potent than urban street PM in causing A549 cell DNA strand breakage and oxidation, that the involvement of Fe was greater for underground PM than for urban PM and that, while over half of the effect on DNA oxidation by street PM was due to water-soluble PM components, only a small proportion of the activity of underground PM was water-soluble [40]. They also used x-ray diffraction to study the crystal structure of the PM, showing that the majority of underground PM Fe was in the form of magnetite (Fe_3_O_4_), while haematite (Fe_2_O_3_) was prevalent in urban PM. This is potentially important, because while Fe in haematite exists purely in the ferric Fe^3+^ (Fe(III)) form, Fe in magnetite exists as a mix of ferrous (Fe^2+^) and ferric Fe (Fe(II,III)). Generation of reactive oxygen species (ROS) by PM is one of the key mechanisms by which PM is thought to have its effects, and this requires an electron donor to reduce molecular oxygen to superoxide, superoxide to peroxide, and peroxide to hydroxyl radical [43]. This last step is the Fenton reaction, catalysed especially efficiently by Fe, and shown in Karlsson’s study to be involved by the effect of addition of H_2_O_2_ resulting in a large increase in DNA oxidation with PM but not in control cultures. Because the oxidation of Fe(II) to Fe(III) liberates the electron required for generation of ROS, ferrous Fe is much more able to generate ROS, and thus magnetite is a potent generator of ROS, whereas haematite is not. This is, however, complicated by the observation that dissolved ferric Fe can be reduced back ferrous Fe by antioxidants present in lung lining fluid, at least in vitro [44], given that the lung lining fluid presents a potent reducing environment [45, 46]. Similar to the Stockholm underground, a predominance of ferrous Fe has been noted in the Shanghai and Seoul underground systems [9, 47]. The predominance of magnetite is not universally observed, however. Querol and colleagues found predominance of haematite in the Barcelona underground, with only a small fraction of magnetite, which has also been noted for the Budapest underground [3, 48]. However, in a discussion of their findings of the predominance of magnetite and maghaemite (γ-Fe_2_O_3_ with a different crystal structure to α-Fe_2_O_3_ in haematite), Jung and colleagues assert that while magnetite and haematite can be differentiated by spectroscopic techniques, this is not possible for magnetite vs. maghaemite [49]. The implication of this is that ferrous and ferric Fe cannot be accurately distinguished by x-ray spectroscopy if the ferric Fe is in the form of maghaemite rather than haematite.

The fact that more of the activity was water soluble in urban PM than underground PM is possibly due to a greater proportion of Fe/other transition metals in urban PM than in underground PM being in the form of water-soluble salts (e.g. sulphate (SO_4_^2−^)), thus increasing the effective metal ion concentration, which in insoluble metal/metal oxides is simply a function of particle surface area. Increased water solubility may modulate the toxicity of these particles, although it is not clear whether it would increase or decrease.

The 2006 paper by the same authors compared Stockholm underground PM_10_ to PM from wood burners (total PM) and tyre wear simulators (PM_10_, with one additional PM_2.5_ sample) representing different types of burner/fuel and road, respectively, and also street PM_10_ collected in a Stockholm city centre street [41]. This showed different effect rankings by endpoint: by comet assay, DNA damage in A549 cells was significantly higher with underground PM_10_ than any other PM tested, while underground PM_10_ induced only relatively small increases in release of TNFα (significant) and IL-6, IL-8 (both non-significant) from human monocyte-derived macrophages, compared to street PM. The release of IL-8 in this study was lower for underground PM than seen by Seaton et al (~ 3-fold vs. ~ 2-fold) [38], but a greater difference for street PM (~ 11-fold vs. ~ 2-fold). The authors suggest that this may be due to the greater concentration of endotoxin in the street PM, to which cultured macrophages are sensitive, but cultured epithelial cells are relatively insensitive. This is because macrophages express the lipopolysaccharide co-receptor CD14, whereas epithelial cells, including A549, do not express CD14, and thus unless there is soluble CD14 in the culture medium, epithelial cells tend to respond much less avidly to lipopolysaccharide [50]. Furthermore, the PM in this study was collected on glass fibre filters, which caused a 5–15-fold increase in cytokine release in the blank filter control cultures. Although the authors attempted to correct for this by comparing cytokine release in the presence of PM to that seen in the filter blank-treated cultures, it is possible that the ability of macrophages to phagocytose PM would be impaired to an extent by the presence of glass fibres. Furthermore, it is unclear whether the inflammatory state clearly induced by the presence of these fibres would have an equal effect on the response to each different PM type.

The same authors performed similar experiments in a 2008 study, but examining different endpoints, and incorporating chemically homogenous PM into the work alongside environmental PM, here in A549 cells [42]. Stockholm underground PM_10_ caused an approximately 4-fold increase over control in mitochondrial depolarisation (similar to wood and diesel PM, greater than street PM and tyre/roadwear PM) and intracellular ROS as measured by dichlorofluorescein (DCF) fluorescence, being the only PM type which increased ROS. To examine the mechanism of these effects, the activity of underground PM was compared to that of magnetite, the principal Fe component of the underground PM. Underground PM increased DNA structural damage, measured by single strange breaks and alkaline labile sites, and DNA oxidative damage, assessed by the number of recognition sites for the enzyme formamidopyrimidine DNA glycosylase (FPG), which repairs oxidatively damaged DNA. Conversely, magnetite particles had only a small effect (trending towards significance) on the former, and none on the latter. These effects were then shown to be ascribable to non-water−/non-citrate-soluble underground PM components, and also more genotoxic than haematite particles or Cu-Zn particles, which were also observed in the underground PM samples. Genotoxicity could not, however, be explained by the main component (magnetite), by water-soluble metals, or by intracellular mobilized Fe. The authors suggest that factors responsible for the effects of underground PM may include surface Fe coordination, microcrystal morphology, and surface Fe ion arrangements. However, there is also evidence that the combination of Fe and Cu, as found in underground PM, can be especially potent in the generation of hydroxyl radicals. While Fe-driven Fenton chemistry is critical in the generation of the hydroxyl radical from peroxide, Cu may be more efficient in generation of other ROS [51, 52]. Thus, Fe in a mixture with a lesser amount of Cu may be the most efficient for overall driving of ROS generating processes – it is perhaps important to note that this is generally what is found in underground PM. This would explain why insoluble metal PM in isolation or in a mixture which is not dissimilar to underground PM (e.g. magnetite, haematite, or Cu-Zn PM) cannot replicate the effects of underground PM.

While this series of papers clearly shows the potential effects of underground PM, it does not provide any conclusive evidence of why the underground PM is toxic, nor that it is necessarily more toxic than other PM types, at least on an equal-mass basis. The authors also suggest that the shard-like shape of a portion of the PM may endow the PM with pro-inflammatory potential. However, although this is a noted feature of the macrophage response to PM which are particularly larger in one dimension than another (i.e. have a high aspect ratio), whereby phagocytosis begins but is unable to complete leading to “frustrated phagocytosis” with consequent release of inflammatory cytokines [53, 54], to our knowledge there is no published evidence that such an effect occurs in epithelial cells.

The study of Lindbom et al (2006) illustrates the difference in responses to PM_10_ of different cell types [55]. Data from monocyte-derived macrophages showed that underground PM was poor at eliciting release of IL-6, IL-8, and TNFα compared to two types of roadwear PM and water and methanol extracts of diesel PM. However, underground PM was more effective than any other PM type in eliciting TNFα release from bronchial epithelial cells (BEAS-2B), while no PM was able to elicit cytokine release from RPMI2640 nasal epithelial cells. Similar experiments in RAW 264.7 macrophages found that street PM_10_ tended to be considerably more inflammogenic in terms of IL-6 and TNFα, although underground PM_10_ elicited a greater release of arachidonic acid, indicating the potential for a greater effect via the eicosanoid pathway [56]. However, underground PM_10_ generated a significantly greater response than did street PM_10_ in terms of various measures of ROS, either directly (by cell-free oxidation of dithiothreitol; DTT) or indirectly (by measurement of thiobarbituric acid-reactive substances as a proxy for lipid peroxidation). In general, granite and quartzite pavement wear particles elicited less response over all outcome measures than did the street and underground PM_10_.

The paper by Bachoual et al (2007) is interesting and unusual in that it compares two underground PM_10_ types – those from the Paris Metro, which uses rubber/pneumatic tyres and “wooden” brakes (PM_10_ Fe = 42%, Mn = < 1%), and those from the suburban Réseau Express Régional (RER) system, which uses metallic components (PM_10_ Fe = 61%, Mn = 7%) [57]. Carbon black, titanium dioxide (TiO_2_), and diesel exhaust PM (DEP) were used as comparators. No source-specific difference in cell death was seen, while the two railway PM sets were the most effective at inducing release of MIP-2 and TNFα from RAW 264.7 murine macrophages, within 3 and 8 h, respectively, and persisting until at least 24 h. Conversely, none of the particles tested induced increased mRNA expression of matrix metalloprotease-(MMP) 2 or 9, and all induced MMP-12 to a roughly equivalent extent. The Fe chelator desferrioxamine (DFX) reduced release of TNFα by RER PM by ~ 50%, but had little effect on the response to Metro PM, while no effect on MIP-2 response was seen with DFX, suggesting differential release pathways for these two cytokines. In mice intratracheally instilled with PM at 0.22–4.48 mg/kg body weight, RER PM but not carbon black or DEP caused increased BAL protein, used as a marker of airway epithelial leakage/damage, with increased BAL total cell and neutrophil percentage. These increased cell counts were seen to a lesser extent with DEP, but not seen following carbon black. Similarly to the in vitro tests, RER PM but not carbon black or DEP induced increased TNFα and MIP-2 release in mice (measured in BALF) within 8 h, and an increase in expression of MIP-12 but not MIP-2 or MIP-8. RER PM also induced increased expression of the antioxidant gene haemoygenase-1 (HO-1), one of the most commonly used markers of antioxidant response to oxidative stress, which was not noted with carbon black or DEP. Therefore, this study suggests that PM from the Paris underground is generally more inflammatory then the other PM types tested, and a portion of this activity derives from the metallic nature of RER PM which was not seen in Metro PM. However, there is also a significant component of the effect of underground PM which does not appear to be related simply to the Fe content of the PM. The authors also suggest that the RER environment is more worthy of investigation on account of its higher PM concentration (361 μg/m^3^ vs. 68 μg/m^3^ in the Metro).

The organic composition of underground PM_10_ is rarely studied, probably on account of the general consensus that the principal sources of organic PM, such as road vehicle exhaust emissions and combustion, contribute little to the underground PM load. However, this was the focus of a study by Jung et al in which the organic extract of underground PM_10_ was able to elicit significant cell death in Chinese hamster ovary (CHO-K1) cells, but not BEAS-2B bronchial epithelial cells [58]. The organic components, and their metabolic breakdown products, were shown to be able to induce micronucleus formation, indicative of DNA damage, and DNA strand breakage in both cell types, and this could be ameliorated by scavengers of superoxide, peroxide, and hydroxyl radical. GC-MS-MS analysis of the organic extract showed the presence of ten of the sixteen US Environmental Protection Agency (EPA) criteria carcinogenic PAHs, although analysis of cytochrome p450 1A1 (CYP1A1) expression, which is usually significantly upregulated by the presence of such PAHs through the aryl hydrocarbon receptor [59], suggested that this underground PM_10_ was not sufficiently PAH-rich as to be able to upregulate CYP1A1 mRNA expression.

The study of Loxham et al in 2015 observed similar effects to the above studies, with PM concentration-dependent increases in IL-8 release and ROS generation, along with upregulation of HO-1 [60]. However, the study was unusual in using primary cells differentiated at the air-liquid interface on Transwell culture membranes. This facilitates formation of functional cilia and mucus-secreting goblet cells, with consequent apical mucous layer, and thus is more representative of the in vivo airway epithelium than is a standard monolayer culture [61]. Underground PM (from a European mainline underground station) was nonetheless able to cross the mucous barrier and exert the above effects, as well as entering cells. This study is also unusual in examining the ultrafine fraction of underground PM, which is generally neglected by other studies. This fraction was found to be as Fe/metal-rich as the other fractions and generated a larger ROS and IL-8 response than the larger size fractions on an equal PM concentration basis [8, 60]. While the ultrafine fraction (44 μg/m^3^) was present in a lower mass concentration than the coarse and fine fractions (180 and 71 μg/m^3^, respectively) in the air of the underground station, this should be seen in the context of the overall greatly raised PM concentration underground, and thus the exposure to UFPM underground is of potential importance to the health impacts of underground railway air pollution.

Spagnolo et al considered PM_10–2.5_, PM_2.5–1_, PM_1–0.5_ and PM_0.5–0.25_ collected at an unnamed underground platform, underground/intermediate commercial area, and outdoor site [62]. By MTT assay, the platform PM was more cytotoxic to H727 bronchial epithelial carcinoma cells than the commercial underground area PM (which showed no significant cytotoxicity), but the three smallest outdoor fractions were the most cytotoxic of all. In contrast to the study of Loxham et al [60], larger PM fractions were better generators of ROS than the smaller fractions at all three sites, although the PM_0.5–0.25_ fraction of platform PM nonetheless generated significantly increased levels of ROS, which was not the case for the same smallest fraction of intermediate or outdoor PM. Levels of ROS generation were strongly correlated with a panel of metals (Mn, Cr, Ti, Fe, Cu, Zn, Ni, Mo) typically found elevated in underground stations PM, although this study did not undertake any mechanistic work to investigate causality of these relationships.

It has been suggested that a key mechanism by which PM exerts toxic effects is through the generation of ROS, and therefore assays which monitor the depletion of antioxidants, as a proxy for the ability of PM to oxidise biomolecules, termed “oxidative potential” (OP), may be useful in measuring potential toxicity of PM [63–65]. Moreno et al (2017) looked at the ability of PM collected from a range of different underground railway locations to deplete ascorbic acid and uric acid and to oxidise glutathione, all of which are measures of the OP of PM [66]. Other measures of OP have been evaluated, such as electron spin resonance and oxidation of dithiothreitol, but ascorbic acid in particular has been shown to be responsive to the typical components of underground PM [67]. The Moreno study found that PM_2.5_ mass was not significantly correlated with OP, and Fe was significantly negatively correlated with ascorbic acid depletion. Conversely, OP was linked to PM Cu, As, Mn, Zn, and Ba concentrations. While it has previously been suggested that in this type of study glutathione (GSH) is not susceptible to the presence of Fe, ascorbic acid depletion does seem to be [63]. Thus, one explanation for the lack of effect of Fe in this study is that it exists in a non-redox-active form such as haematite. The authors suggest that these correlations, along with the compositions of the most oxidative PM samples, indicate that a significant source of PM OP derived from brake and catenary wear. They also note that the lowest OP is found in the newest station which has platform-edge doors (PEDs), which have been seen to reduce platform PM concentrations [49, 68].

Similarly, Gali and colleagues investigated the redox characteristics of PM from personal samplers of passengers making journeys on above-ground and below-ground routes of the Hong Kong underground (including time on the train and waiting on the platform), and compared this with PM collected from journeys on an overground train route, bus, and ambient PM [69]. This study was unusual in that the underground concentration of coarse and fine PM was lower than found in above ground train routes, with the lower concentrations underground attributed to the use of platform-edge doors, while the underground PM samples were also less metal-rich than seen in other studies, for example the Fe concentration of PM_2.5_ being ~ 0.2% by mass. Coarse underground PM was seen to be more potent in reducing cell viability than coarse PM from above ground or bus journeys, but for fine PM, there was little difference across samples, although fine PM was generally more cytotoxic than coarse PM. On a mass basis, underground PM was generally more potent in generating extracellular ROS than overground train and bus PM, and underground coarse PM was slightly more potent than underground fine PM in the generation of intracellular ROS, although there was general equipotency in terms of intracellular ROS generation by the fine PM samples. However, when considered on the basis of airborne PM concentration, by volume, overground train PM was a more potent generator of ROS than underground PM. In underground PM, intracellular ROS generation was associated with mass concentration of Al, Ba, Cu, Mn, Mo, Ni, V, Mg, and Na. Interestingly, there was no correlation with Fe concentration, in agreement with Moreno and colleagues [66]. Furthermore, ROS generation by underground PM was significantly lower than seen in PM from urban sites in a previous study by the same authors [70]. This suggests that underground PM metals may be less soluble and therefore less bioavailable in underground PM, and this limiting the ability of underground PM to generate intracellular ROS.

#### Summary

These studies are summarised in Table 2. Studies which have examined release of inflammatory cytokines from cell cultures exposed to PM (in vitro) indicate that underground PM is able to elicit release of the commonly studied inflammatory cytokines, including IL-6, IL-8, and TNFα. Where studies have directly compared the effects of underground PM to other types of PM, it seems that underground PM is more potent in this regard than carbon black or TiO_2_, and also roadwear particles, but less so than urban PM. The increased pro-inflammatory potency of urban PM may relate to the increased concentration of PAHs or other organic species, or of endotoxin/lipopolysaccharide, the latter evidenced by a trend for heightened macrophage, but not epithelial, pro-inflammatory responses to street/urban PM compared to underground PM. It is also possible that the greater PNC of urban PM vs. underground PM plays a role in this effect, given the suggested importance of PNC in driving particle toxicity and the known effects of UFPM and nano-sized PM [39, 71].

**Table 2:** In vitro studies of the toxicology of underground railway particulate matter

Author	Publication Year	Underground	[Airborne PM] (μg/m^3^ unless stated)	Underground PM Composition	Comparator PM	Model	Exposure Conc/Time	Findings
Seaton [38]	2005	London, UK	PM_2.5_ = 270–480; PNC = 14,000-29,000/cm^3^	PM_2.5_: Fe = 64–71%; Cr = 0.1–0.2%; Mn = 0.5–1%; Cu = 0.1–0.9%; quartz = 1–2%	Urban PM_10_; TiO_2_; welding fume	A549	PM_2.5_ 1–100 μg/ml, 8–24 h	Underground PM_2.5_ caused concentration-dependent increase in IL-8 release, LDH release, plasmid damage.
Karlsson [40]	2005	Stockholm, Sweden	Not stated	PM_10_: Fe = 39% (mainly Fe_3_O_4_); Si = 6%; Al = 3%; Ca = 1%; Cu < 1%; Ba< 1%; Mn < 1%	Urban street PM_10_	A549	PM_10_ 9–70 μg/ml (5–40 μg/cm^2^), 4 h	Underground PM_10_ more genotoxic and oxidative-stress inducing than urban PM_10_.
Karlsson [41]	2006	Stockholm, Sweden	Not stated	Not stated (may be same as [40])	Wood boiler PM; tyre wear PM_10_/PM_2.5_; urban PM_10_	A549; monocyte-derived macrophages	PM_10_ 70 μg/ml (40 μg/cm^2^), 4 h	Underground PM_10_ induced more DNA damage in A549 cells than other PM tested. In macrophages, urban PM_10_ was most potent inducer of inflammatory mediator release.
Karlsson [42]	2008	Stockholm, Sweden	Not stated	″	Wood boiler PM; tyre wear PM_10_, urban PM_10_; diesel PM; Fe_3_O_4_; Fe_2_O_3_; CuO; Cu-Zn	A549	PM_10_ 35–70 μg/ml (20–40 μg/cm^2^), 2–8 h	For mitochondrial depolarisation by PM_10_, DEP > underground = wood>street>tyre. Underground PM_10_ most potent ROS generator, and increased FPG sites and DNA damage more than Fe_3_O_4_, Fe_2_O_3_, CuO, Cu-Zn.
Lindbom [55]	2006	Stockholm, Sweden	PM_10_ = 469; PM_2.5_ = 258	Predominantly Fe, with some Si, Ca, Ba, Cu	Roadwear PM_10_; street PM_10_; DEP	Monocyte-derived macrophages; RPMI 2650 nasal epithelial cells; BEAS-2B	PM_10_ 10–500 μg/ml, 18 h	Underground PM_10_ was less potent in eliciting IL-6, IL-8, TNFα release from macrophages, but most potent in eliciting their release from BEAS-2B.
Lindbom [56]	2007	Stockholm, Sweden	″	″	Roadwear PM_10_, street PM_10_	RAW 264.7 macrophages	PM_10_ 1–100 μg/ml, 18 h	For inflammatory mediator release by PM_10_, street>underground>roadwear. For arachidonic acid release and measures of oxidative stress (DTT, TBARS), underground>street>roadwear.
Bachoual [57]	2007	Metro and RER, Paris, France	PM_10_ Metro = 67; RER = 3609	PM_10_ Metro: Fe = 41.8%; Mn < 1%; Ca = 1.25%; Cu = 1.2%; S = 2.2%; Si = 1.45%; PM_10_ RER: Fe = 61%; Mn = 7%; Ca = 0.2%; Cu = 0.45%; S = 1.95%; Si = 1.8%	Carbon black; TiO_2_; DEP	RAW 264.7 macrophages; C57BL/6 mice	PM_10_ RAW 264.7: 0.05–50 μg/ml (0.01–10 μg/cm^2^), 3–24 h; Mice: 0.22–4.48 mg/kg (5–100 μg/mouse), 8/24 h	RAW 264.7: underground PM_10_ sets elicited most MIP2 and TNFα release. DFX reduced TNFα release by RER but not Metro PM_10_. Mice: RER PM_10_ but not CB or DEP induced release of TNFα and MIP2, and HO-1 expression.
Jung [58]	2012	Seoul, South Korea	PM_10_ = 34; PM_2.5_ = 4.5	Not stated	None	CHO-K1; BEAS-2B	1.6–100 μg/ml organic extract of PM_10_	Underground PM_10_ induced significant cell death in CHO-K1, but not BEAS-2B cells. DNA micronucleus formation and strand breakage by underground PM_10_ inhibited by ROS scavengers.
Loxham [60]	2015	Mainline underground station, Europe	PM_10–2.5_ = 180; PM_2.5_ = 71; PM_0.18_ = 44	PM_10–2.5_: Fe = 32.1%, Cu = 1.68%; Mg = 1.63%; Ca = 1.52%; PM_2.5_: Fe = 28.4%; Cu = 1.41%; Mg = 2.12%; Ca = 1.52%; PM_0.18_: Fe = 32.9%; Cu = 1.71%; Mg = 2.56%; Ca = 2.20% (see also [8])	None	16HBE14o-; PBEC	PM_10–2.5_, PM_2.5_, PM_0.18_ 6.25–50 μg/ml (0.6–12.5 μg/cm^2^), 24 h	PM crosses PBEC mucous barrier to cause concentration-dependent release of IL-8 increasing with smaller PM size. ROS generation and HO-1 induction observed, both inhibited by DFX and NAC.
Spagnolo [62]	2015	Not stated	PM_10–2.5_ = 26; PM_2.5–1_ = 13; PM_1–0.5_ = 3.7 μg/m^3^; PM_0.5–0.25_ = 14 μg/m^3^	(All ng/m^3^) PM_10–2.5_: Fe = 545, Ca = 1568, Ba = 122, Cr = 15, Cu = 14; PM_2.5–1_: Fe = 212, Ca = 256, Ba = 96, Cr = 3, Cu = 12; PM1–0.5: Fe = 71, Ca = 58 Ba = 99, Cr = 2 Cu = 4; PM_0.5–0.25_: Fe = 31; Ca = 30; Ba = 99; Cr = ND; Cu = 3	Commercial/intermediate station area PM; outdoor PM	NCI-H727	70 μg/ml, 3/6/24 h	Cytotoxicity: platform PM > intermediate area PM, but smallest fractions of outdoor PM most cytotoxic. ROS generation: larger PM sizes>smaller PM sizes. Correlations between transition metals and ROS generation.
Moreno [66]	2017	Barcelona (six stations), Spain	PM_2.5_ = 33–87 (102 during maintenance activity)	(All ng/m^3^) PM_2.5_: Fe = 8000-34,000, Ca = 500–1300 Cu = 33–331, Mn = 107–301	M120(CB), NIST1648a	Cell-free depletion of ascorbate and GSH	PM_2.5_, cell-free	Antioxidant depletion not associated with PM mass. Antioxidant depletion positively associated with Cu, As, Mn, Zn, Ba, ascorbate depletion negatively associated with Fe
Janssen [67]	2014	Mainline underground station, Europe	PM_10_ = 409; PM_2.5_ = 143	Not stated (see [8] for characterisation of separate samples from same location)	PM_10_ and PM_2.5_: urban background; continuous traffic; stop-go traffic; farm	Cell-free depletion of ascorbate, DTT, ESR	PM_10_ and PM_2.5_, cell-free	Underground PM had greatest oxidative potential of all PM types studied.
Gali [69]	2017	Hong Kong	PM_10–2.5_ = 10 ± 5; PM_2.5_ = 48 ± 13	Data as graph only, Fe ≈ 0.2% (similar to other PM sets in study)	PM_10–2.5_ and PM_2.5_: above ground railway journey; bus journey; ambient site	RAW 264.7 macrophages	10–100 μg PM suspension, 4/24 h	Underground PM_10–2.5_ had greatest negative effect on cell viability. Little difference across PM_2.5_ sets. Mass/mass: underground PM_10–2.5_ was best generator of ROS. Mass/volume: above ground PM was more potent. No association with Fe.

It should also be considered that while in vitro studies tend to test identical mass concentration of PM types, inhaled air contains different PM mass concentrations – underground PM mass concentrations may be several times greater than those above ground, although one cannot necessarily apply a multiplicative correction factor as this would assume linearity of the concentration-response relationship. There is some evidence that such linearity may exist at ambient PM concentrations, but this may break down at PM concentrations found underground, and may also depend on the endpoint and the nature of the PM [72].

Another observation is that underground PM appears to have a greater OP and a greater ability to exert oxidative damage in vitro than urban PM. The observation that underground PM can elicit damage to DNA (through oxidised bases or strand breaks) and lipid (per)oxidation, in terms of production of thiobarbituric acid-reactive substances (TBARS) is important. It is interesting that OP, as measured by the depletion of one or more antioxidants in vitro, appears to be a good predictor of cellular response with respect to certain endpoints in vitro, but that there is much less evidence to link it directly to effects in vivo (for the best example of this over a range of outcome measures, see the series of papers from the RAPTES study (section 3)).

The question arises as to the component(s) of underground PM important for its effect. In this regard, the fact that a number of studies show that Fe chelation/redox inactivation is able to significantly reduce the effects of underground PM indicates that the Fe content of underground PM (generally in the range of 20–60% by mass) is important. This can also be seen where two underground systems in the same city but with different components are studied [57]. However, the finding that magnetite and haematite are unable to replicate these effects also suggests that there may be other important components, perhaps “working” in concert with the Fe-containing species. One possibility is that there are other metals also involved, and maybe even predominating in determining OP, such as brake and catenary wear Cu and Sb [66]. This would explain why Fe chelation can significantly but nonetheless only partially reduce the effects of underground PM, and also why correlations between ROS generation and PM elemental concentrations often highlight the importance of non-ferrous metals. There is also the possibility that organic species may contribute [58], although since these species likely derive predominantly from the outside environment, this may not apply to deep-lying stations with poor air exchange [73, 74]. Notably, the greater effects of underground PM on various markers of oxidative stress and oxidative damage occur despite the PNC of underground PM being lower than that of urban PM, thus strongly implicating a composition component of underground PM, of which transition metals are the most obvious candidate. The ability of metal-rich PM to generate ROS may be of relevance in asthma, where even in the mild form of the disease there is thought to be dysregulated antioxidant defence in the airways [75], and where in vitro evidence suggests that the airway epithelium may be more susceptible to oxidant-induced damage [76].

### (3) RAPTES - risk of airborne particles, a toxicological-epidemiological hybrid study

The RAPTES study was a multi-part study of the effects of exposure to PM from a range of locations in the Netherlands, attempting to link PM source and composition to effect. Sites used in the study were an underground railway station, an urban background site, a farm, three traffic sites (continuous traffic, stop-go traffic, and truck traffic), a harbour, and an area near a steelworks. The underground site had a much higher mass concentration of PM_10_ and PM_2.5_ than other sites (394 and 137 μg/m^3^, respectively), but lower PNC than the road traffic sites. In the coarse PM fraction, mass/volume concentrations of Fe and Cu were approximately two orders of magnitude higher underground than any other site, and Ni one order of magnitude higher, along with elemental carbon (EC) one order of magnitude higher, and EC/organic carbon (OC) ratio higher than any other site. In the fine fraction, underground PM was also more EC rich but had an EC/OC ratio comparable to the traffic sites [77]. Across coarse, fine, and quasi-ultrafine (< 0.18 μm) fractions, underground PM had a greater effect on RAW 264.7 macrophage viability, and coarse underground PM was the most potent coarse PM in eliciting release of TNFα and MIP-2, although underground PM was less active in this regard for fine and quasi-ultrafine fractions compared to traffic PM. Only traffic and steelworks fine and quasi-UFPM elicited IL-6 release. While across all samples there was no association between PM OP and cytokine release, a positive association was seen when underground PM was excluded from correlation analysis, suggesting that underground PM possesses fundamentally different chemistry from the other PM types [78].

Following in vitro studies, five of the sites were used for human exposure studies (underground, urban background, continuous traffic, stop-go traffic, and farm). These studies found associations between F_E_NO, which represents eosinophilic airway inflammation [79], and PM Fe, V, Cu, and water-soluble Ni, and between loss of lung function (by FVC and FEV_1_) and Fe, Cu, and water-soluble Ni, but not PM_10_ mass concentration or OP [80]. In nasal lavage, IL-6 and IL-8 were associated with organic carbon, NO_2_ and endotoxin concentrations. Concentrations of lactoferrin, a metal-binding protein with both pro- and anti-inflammatory properties, were associated with the high PM metal concentration found at the underground site [81]. Plasma concentrations of cardiovascular risk markers C-reactive protein (CRP), fibrinogen, von Willebrand factor (vWF), and tissue plasminogen activator/plasminogen activator inhibitor-1 complex, as well as platelet count, were associated with PM OC, nitrate (NO_3_^−^), and SO_4_^2−^, although the latter two may reflect increased bioavailability of metals rather than being the causative species per se [82], while thrombin generation in blood taken from exposed volunteers was associated with PM NO_3_^−^ and SO_4_^2−^, as well as NO_2_ concentration [83]. After 2 h exposure, there was an increase in circulating neutrophil count, while 18 h after 5 h exposure there was an increase in circulating monocytes, associated with PM_10_ and PM_2.5_ mass concentrations, EC, and PM OP [84]. These associations were, however, driven by underground exposure, and were not present when underground exposures were excluded from analyses, further emphasising the atypical nature of underground PM. Because of the consistently higher concentration of certain PM characteristics at the underground site compared to the other study sites, it was not possible to determine the characteristics driving this response, but it is notable that most factors other than PM OP were excluded.

Over the RAPTES studies (summarised in Table 3), it is noteworthy that different endpoints were responsive to different PM characteristics. Furthermore, it can be seen that short term (2 h) exposures were sufficient to induce measurable changes in vivo, which may be important in understanding the potential effects of underground air exposure on passengers, as well as workers who may be exposed for longer periods. While it was noted that some, although not all, of these endpoints were especially responsive to underground PM, it is also notable that in several cases, correlations between PM characteristics and endpoint were driven by the presence/absence of underground PM in the analyses, implying that underground PM represents a distinct type of PM compared to the other PM types analysed. However, the endpoints measured were not always associated with factors enriched in underground PM; in several cases, outcomes were associated with organic carbon concentrations. The degree of difference between underground PM and the other PM samples renders it difficult to delineate the specific components of underground PM responsible for its effects.

**Table 3:** Papers arising from the RAPTES series of studies

First Author	Year (sampling/exposure)	Underground	[Airborne PM] (μg/m^3^ unless stated)	Underground PM Composition	Comparator PM	Model	Exposure Conditions	Sample Size	Findings
Steenhof [78]	2011 (June 2007–February 2008)	Mainline underground station, Europe (same as [8, 60])	PM_10–2.5_ = 58; PM_2.5–0.18_ = 38; PM_0.18_ = 83; PNC = 39,000/cm^2^	Fe = 30.5%; Cu = 2.7%; Zn = 1.2%	Urban background; continuous traffic; stop-go traffic; truck traffic; farm; steelworks; harbour	RAW 264.7 macrophages	6.25–100 μg/ml (3.68–58.8 μg/cm^2^), 16 h	N/A	All sizes of underground PM were most potent in reducing cell viability. Coarse underground PM most potent inducer of TNFα and MIP-2 release, otherwise traffic PM generally more pro-inflammatory.
Strak [80]	2012 (March–October 2009)	″	PM_10_ = 394; PM_2.5_ = 140; PM_10–2.5_ = 252	Fe = 154 μg/m^3^; Cu = 7 μg/m^3^; Ni = 68 ng/m^3^; V = 25 ng/m^3^	Urban background, continuous traffic, stop-go traffic, farm	In vivo human	5 h	31	F_E_NO was associated with PM Fe, V, Cu, and water soluble Ni, and loss of FVC and FEV_1_ with Fe, Cu, and water soluble Ni. No association with PM_10_ mass or OP.
Steenhof [81]	2013 (March–October 2009)	″	″	″	″	″	″	″	OC, NO_2_, and endotoxin associated with nasal lavage IL-6 and IL-8. Lactoferrin associated with underground PM metal.
Strak [82]	2013 (March–October 2009)	″	″	″	″	″	″	″	Plasma CRP, fibrinogen, VWF, tPA-PAI-1, platelet count associated with PM OC, NO_3_^−^, SO_4_^2−^.
Strak [83]	2013 (March–October 2009)	″	″	″	″	″	″	″	Ex vivo blood thrombin generation associated with PM NO_3_^−^ and SO_4_^2−^.
Steenhof [84]	2014 (March–October 2009)	″	″	″	″	″	″	″	Increase in circulating monocytes associated with PM_10_ and PM_2.5_ mass, EC, and PM OP, mainly driven by atypical characteristics of underground PM.

### (4) In vitro-in vivo extrapolation for risk assessment

A small number of studies have attempted to quantify health impacts of underground PM from data generated in in vitro experiments. The group of Constantinos Sioutas assessed the health effects of PM in the Los Angeles underground in three studies [85–87]. In the first, they compared the composition and ROS generating capacity of coarse and fine PM from an underground railway and an overground railway, collected from stations and on trains to represent real-life exposure, and an ambient site at University of Southern California [85]. PM_10_ concentrations were highest in the underground station, but this was driven by fine PM mass concentration, whereas coarse PM underground was higher than the overground station but similar to ambient concentrations. In terms of composition, the most notable difference was enrichment of Fe in the underground samples compared to overground and ambient samples along with enrichment of non-crustal species such as Mn, Cr, Co, Ni, Cu, Ba, Mo, Cd, and Eu, with the enrichment more pronounced in the fine fraction than the coarse fraction on both PM mass/mass and mass/volume concentration measures. This concentration enrichment was attributed to the specific sources of these elements underground and the enclosed environment of the underground, respectively. Secondary ions and organic carbon species underground were suggested to have derived from outdoor sources. Crustal species concentrations were similar in coarse PM underground and above ground, but there was suggested to be an additional source of Al and Ca in fine PM underground. Correlation analyses suggested that Al and Ca, along with the aforementioned non-crustal species, likely derived from a single railway-specific source present above and below ground, as there was a generally high correlation between these elements. However, the authors also suggest additional other sources for Ba (brake wear), Cu (sources not suggested, although may be electrical contact components), and Zn (may originate from above-ground vehicular emissions). As noted in other studies earlier, these elements generally exhibited lower water solubility in underground PM than in overground or ambient PM, across both coarse and fine PM. When taking airborne PM mass concentration into account, Fe and Ba were the only water-soluble components enriched in underground PM compared to the other two sites. Across all sites, ROS generation in DCF-loaded PM-exposed macrophages was strongly correlated with water-soluble Fe, Ni, Cr, Cd (which is not redox active) and organic carbon. Further analysis indicated that, across all sites and PM types, 94% of ROS generating variability could be explained by water soluble Fe and organic carbon concentrations. Fine PM across all sites possessed greater ROS generating capacity than coarse PM. On a PM mass basis, underground coarse PM generated slightly more ROS than the other PM samples, while in the fine fraction overground railway PM generated more ROS than underground and ambient PM, which were approximately equipotent. When taking airborne concentration into account, on a PM mass/volume basis, coarse and fine PM at the underground site generated more ROS than the overground rail and ambient sites, but the magnitude of this difference was not as great as might have been expected if only the elemental concentration of PM samples were considered.

A second study by the same group compared PM on the underground and overground lines with two roads, one with low heavy goods vehicle (HGV) usage and one with high HGV usage [86]. Analysis of the potential lung cancer risk due to PAH exposure was performed, and suggested that the lung cancer risk was highest from the HGV-high roadway, on account of the relatively higher concentrations of PAH. While the underground line had the lowest PAH concentration, it was suggested that the overground rail line, which had a PAH concentration almost as low as the underground line, along with a lower PM load and metal concentration than the underground line, may represent the safest route with its combination of low PAH and low PM. The US EPA recognises 16 PAHs as carcinogenic and requiring of monitoring [88], and it was on this basis that the authors initially restricted their calculations to this group.

However, it is also recognised that PAH are not the only carcinogen/potential carcinogen in airborne PM. Therefore, the same group performed a further study to evaluate the effects of metals within underground PM_2.5_, both in terms of carcinogenicity and non-carcinogenic toxicity, and set these effects within the context of the carcinogenicity from arising from PAH exposure [87]. This was done by measuring concentrations of organic carbon and metals in underground and overground railway systems, as well as the two roadway classes in the previous study, and deriving the carcinogenicity and toxicity potentials from values established by the US EPA and the California Office of Environmental Health Hazard Assessment. Notably, the contribution to increased cancer and non-cancer risk was found to be greater for metals especially enriched in underground PM_2.5_ compared to the contribution for PAHs enriched in traffic-derived PM. This was especially true for Cr, which was assumed to exist wholly as hexavalent Cr(VI), which is highly carcinogenic [89], as well as Ni, and Cd. This carcinogenicity is due to the ability of Cr(VI) to enter cells as a result of the similarity of the hexavalent Cr oxyanion (CrO_4_^2−^) to phosphate (PO_4_^2−^) and SO_4_^2−^ anions and the relatively non-selective anion uptake channels used [89, 90]. Cr(VI) is then reduced intracellularly to Cr(III), which forms DNA adducts. For non-cancer risk (termed the “hazard quotient”) the increased risk of underground PM exposure was driven by Cd, Cr, Ni, and Mn. The cancer and non-cancer health risks were approximately one order of magnitude higher for underground exposure compared to roadway exposure. The authors note that while the permissible exposure levels set by the US Occupational Health and Safety Administration were not exceeded, the excess lifetime cancer risk (ELCR), at 10^− 5^ over a lifetime of exposure, is an order of magnitude greater than the permitted value of 10^− 6^. This is driven primarily by Cr concentrations in the underground, which were found to be 100–1000 times greater than ambient concentrations. However, the conclusions of this paper rely partly on the key assumption that underground Cr in PM_2.5_ exists in the Cr(VI) form on account of its high temperature formation – this was not verified, although a similar assumption has also been made elsewhere [91].

Cao and colleagues monitored PM_2.5_ and NO_2_ concentrations in five railways stations in Suzhou, China, with different characteristics such as overground, underground, urban centre, and industrial area [92]. They found increased concentrations of PM_2.5_ and decreased NO_2_ in underground stations compared to overground stations, and increased PM_2.5_ in underground stations in urban areas compared to those in green areas. The observed average underground platform PM_2.5_ concentration during rush hour periods of 265 μg/m^3^ is higher than in most other studies. Furthermore, underground PM_2.5_ and NO_2_ concentrations increased in rush hour. PM_2.5_ concentrations were lower in carriages than on platforms, a finding attributed to the use of in-carriage air filters. Underground PM_2.5_ in the summer was significantly lower than in the spring, suggested to be due to increased humidity in the summer. The authors then attempted to derive inhaled dose and use this value, along with underground PM_2.5_ and NO_2_ concentrations and journey numbers to calculate DALYs, arriving at a value of 6390 DALYs in 2015, equating to 375 premature deaths, or 1% of the total deaths in the city as a result of underground air pollution exposure. However, the authors did not consider a similar calculation for above-ground exposure, nor did they account for the unusual chemistry of PM_2.5_ in the underground railway, or the likelihood that underground passengers may represent a relatively healthy, and therefore less susceptible, subgroup of the population. Thus, such figures cannot be taken as indicative across a whole population.

#### Summary

These studies (summarised in Table 4) provide evidence to support the assertion that underground PM should not be regarded as simply an Fe-rich particle, but that consideration should be given to other metal constituents which may also play a role in PM toxicity, as well as the solubility of the metals, which may be lower in underground PM than in ambient PM [85]. This work illustrates that the carcinogenic and non-carcinogenic effects of non-ferrous metals in underground PM may outweigh the effects of the PAH in the heavily trafficked roads. This also lends further support to the studies featured in Section 2, which are suggestive of important roles for non-ferrous metals in the toxicity of underground PM [66, 69]. While the specific risk factors, attributed disability adjusted life years (DALYs), and similar derived values rely on a number of assumptions, such studies serve to highlight the potential effects of underground PM exposure on large populations, and illustrate the diversity of potential toxicants within underground PM, thus emphasising the importance of considering the totality of PM composition, rather than solely the predominant components.

**Table 4:** Studies using in vitro*-*in vivo extrapolation for risk assessment of exposure to underground railway air pollution

First Author	Year	Location	[Airborne PM] (μg/m^3^)	PM Composition	Comparator PM	Model	Findings
Kam [85]	2011 (sampling May–August 2010)	Los Angeles, USA	PM_10–2.5_ = 11 ± 2; PM_2.5_ = 33 ± 1	PM_10–2.5_: Fe = 27%; PM_2.5_: Fe = 32%	PM_10–2.5_ and PM_2.5_: overground train journey; ambient	Alveolar macrophage	Underground PM enriched in Fe, Mn, Cr, Co, Ni, Cu, Ba, Mo, Cd, Eu, especially in PM_2.5–0.1_. In terms of water-soluble elements, only Fe and Ba were higher in underground PM. For ROS generation, underground>overground>ambient, but difference small.
Kam [86]	2013 (sampling May–August 2010)	″	″	″	PM_2.5_: overground railway; HGV-heavy and HGV-light freeways; stop-go road	N/A	On the basis of airborne PAH concentration, lung cancer risk was: HGV-heavy road>HGV-freeway>stop-go road>overground railway>underground railway
Lovett [87]	2018 (sampling May–August 2010)	″	″	″	″	N/A	Extending [86] to also take metals into account, total hazard quotient from PM exposure greatest on the underground, mainly due to Cr(VI). Overground railway has lowest hazard.
Cao [92]	2017 (measurement March–August 2015)	Suzhou, China	PM_2.5_ regular hours: underground platform = 198 (range 86–351); carriages = 60 (45–121); PM_2.5_ rush hours: platform = 265 (112–365); carriages = 79 (75–145)	Not stated	4 underground stations, 1 above ground station	N/A	PM_2.5_ underground stations>overground, especially in urban vs. green areas. Underground PM_2.5_ summer>spring. Underground exposure associated with 6390 DALYs = 375 premature deaths = 1% total deaths in the city.

### (5) Studies of the effects of exposure to Fe-rich particles generated by processes such as grinding, polishing, and milling

In addition to studies on the in vivo effects of exposure to underground PM (section 1), there are studies examining the in vivo effects of exposure to PM which, while not originating from an underground station, might be expected to be similar to underground PM in terms of chemical composition, or at least more so than ambient PM. A report by the Institute of Occupational Medicine suggested that welding fume may represent a surrogate for underground PM [38, 93]. However, the majority of the mass of PM on the underground is likely to derive from shearing and abrasion as takes place in steel mills, rather than through vaporisation, which is the case with welding fume generation [94].

These studies may offer insight into the potential effects of underground railway PM, because they feature exposure to PM which is (1) enriched in Fe compared to that above ground, and (2) at concentrations closer to those which might be found in a typical underground station compared to urban PM. Furthermore, this enrichment is not simply a fume-derived process as is the case for welding fume which, on account of its generation method, results in large numbers of particulates of a small diameter, with consequent high PNC and total particle surface area. In terms of effects on underground staff, it is reasonable to assume that underground drivers and steel mill workers would likely be composed of mainly young and middle aged workers, predominantly male, although the latter may not apply to staff elsewhere on the underground such as ticket office and platform staff.

Studies performed near a Canadian steel mill observed associations between various airborne metals and markers of adverse cardiac effects [95], having previously observed that spending 8 h periods near to a steel mill was associated with small decreases in lung function [96]. However, airborne concentrations of Fe and other transition metals near the steel mill were at least 2 orders of magnitude lower than might be seen in an underground station, and no association of health effects with Fe was noted. Furthermore, these findings were not consistent with wind direction from the steel mill, and confounded by associations with SO_2_ and NO_2_ concentrations, which was also the case for the observed association with decreased HRV in a follow-up cross-over study [97]. A follow-up study did find an association between the increased UFPM PNC at the near-steel mill site and urinary 8-isoprostane with a 2 day lag, and also between 8-OHdG and NO_2_ and NO with a 1 day lag, suggesting a delayed effect of steel mill emissions on urinary biomarkers of oxidative stress [98], but this does not necessarily implicate Fe in the association.

Studies on workers at an Italian steel mill showed exposures to PM_10_ and PM_1_ similar to those which might be expected in an average underground station, although with a notably lower proportion of Fe (mean 32 μg/m^3^ in a mean airborne PM_10_ load of 233 μg/m^3^) [99]. 17/88 extracellular vesicular microRNAs evaluated in blood were significantly correlated (16 positive, 1 negative) with PM and metal exposure [99], principally miR196b which is linked to poor prognosis in a number of cancers, with a role in epithelial-to-mesenchymal transition and thus metastasis [100], and also a potential role in insulin biosynthesis [101]. Previous studies at the same site found changes in blood microvesicle miRNAs and upregulation of miR-302c and miR-128 after 4 days of work following a 2 day rest period [102], and also associations between PM_10_, PM_1_, and Zn concentrations and increased blood endogenous thrombin potential (ETP), methylation of NOS3 (nitric oxide synthase-3), and decreased EDN1 (endothelin-1) methylation (Zn only) [103]. As length of employment increased, irrespective of donor age, there was increased demethylation of histone 3 lysine 4 (H3K4) and acetylation of histone 3 lysine 9 (H3K9), both associated with Ni and As exposure [104]. All of these results are interesting and suggest the need for future work, but do not suggest any specific health effects of exposure to steel mill PM per se.

Studies using the closure of a steel mill in the Utah valley to investigate PM_10_ concentrations and health outcomes were performed in the 1980s. These showed an 89% increase in hospital respiratory admissions amongst children, and a 47% increase amongst adults, when PM_10_ exceeded 50 μg/m^3^, while PM_10_ concentrations decreased when the steel mill closed [105]. However, it is unclear from this study whether these effects were due to decreased Fe-rich or combustion-related emissions. Water-soluble extracts of ambient total suspended PM (TSP) samples from the local area taken when the steel mill was open were also more able to cause apoptosis, increased BAL fluid cell count, and airways hyperresponsiveness when instilled in rat airways compared to PM samples collected when the mill was closed [106]. Lung injury and inflammatory mediator release in vitro induced by TSP collected nearby in 1982 were reduced when the PM was pre-treated with a chelator [107]. Importantly, effects on cell injury and intracellular signalling in vitro caused by Utah PM could be recapitulated by treating cells with simple mixtures of the most common metals in the PM samples [108]. However, no clear picture has emerged as to which metal is most likely to be particularly relevant to health or if elemental interactions play a role. In this regard, the latter paper above suggests that it is the combination of metals, rather than their individual presence, which is of critical importance in determining outcome.

It is noteworthy that in most cases, these studies find associations of health effects with non-ferrous metals rather than Fe, suggesting that other metals associated directly or indirectly with steel manufacture may drive health effects. Similarly, in several of the papers detailed in this review, these other elements have been suggested to be important drivers of some of the effects of underground PM, and would likely be less enriched in steel mill PM.

Nonetheless, if it is assumed, with caveats, that such steel mill investigations might provide insight into the potential effects of underground PM, their evidence for an effect of underground PM is generally minimal – small changes in respiratory and cardiac function with elements not associated with underground PM, and effects on microRNA and DNA methylation without obvious manifestations in vivo. Therefore, no clear conclusions about the end effects of underground PM can be drawn from these studies, but the potential effects of steel-associated PM do merit further study. In this regard, it is notable that a study of the associations between PM_2.5_ and respiratory and cardiovascular hospital admissions in New York in 2001 and 2002 showed that steel emissions, characterised by Fe and Mn, likely from the World Trade Center construction site, were significantly associated with increased respiratory admissions with 0 and 3 days lag, for asthma and pneumonia, respectively [109]. Conversely, traffic PM was associated with total cardiovascular admissions with 0 days lag, although these extra admissions may have been restricted to vulnerable subgroups who would be less likely to use the underground.

### (6) Other studies of PM metal composition

In addition to work on steel mill exposures, there are many other studies examining the contribution of specific PM_2.5_ components on health outcomes. These are the subject of an excellent review by Morton Lippman [110], which also covers the National Particle Component Toxicity (NPACT) series of studies, and will not be discussed at length here. In short, while it is clear that transition metals are one of the components of PM_2.5_ consistently linked to adverse effects on health following acute exposure, the specific metals implicated are much less consistent across studies. In terms of sources, short-term effects are generally associated with road vehicle and ship emissions (e.g. OC, EC, Cu for traffic, and Ni and V for ship marine oil combustion), whereas longer-term effects are more associated with coal combustion. In ambient exposures, correlation between many of the constituents means that identification of the most toxic specific chemical species, rather than sources, is difficult. This makes extrapolation of the results to underground railways difficult because underground PM is very rarely included in such studies. Thus, it is difficult to determine whether any element present in elevated concentrations underground may pose a specific risk to health based on findings from ambient PM because such an extrapolation requires knowledge of causation rather than simply association, unless the key sources are the same. Even if certain elements in ambient air could be conclusively shown to cause certain health outcomes, these elements may not exist in the same form (e.g. metallic/oxide/sulphate/nitrate), oxidation state, or combination as underground – these factors could potentially influence ROS generating capacity, which is likely an important modulator of toxicity. Furthermore, the mass concentration and exposure rate to PM underground is much different to above ground, which may have consequences for biological response.

## Conclusion

From in vitro studies using cell lines, through in vitro*-*in vivo extrapolation from chemical composition, to controlled short-term exposure studies in vivo, and epidemiological studies of underground railway workers exposed chronically, there are several conclusions which can be drawn about the effects of underground railway PM:

1. Studies which compare airborne PM mass concentrations in underground railways with those above ground generally find increased PM concentrations underground. However, such studies tend to focus on a relatively small number of networks, in cities with relatively low ambient PM concentrations, and older underground railway networks which are less likely to have air conditioning systems installed. Further research is needed to understand whether these findings also apply to newer air conditioned networks in more heavily-polluted cities. Other factors which may increase the extent to which underground PM characteristics and concentration are different from those above ground include station depth, station age, wheel/rail type, air conditioning system, and distance from portal where the trains enter/leave the outside environment.

2. Underground PM is generally transition metal-rich, whether measured per mass of PM or per volume of air. Fe predominates, but there is a general enrichment of steel-associated elements, and also elements associated with train brake wear, electrical components, and lubricants, such as Cu, Sb, and Ba.

3. The proportion of the mass of transition metals in underground PM which is water-soluble is markedly lower than in urban PM. As such, the airborne mass concentration of water-soluble transition metals within underground PM is not necessarily greatly elevated over ambient PM. Consequently, the concentration of bioavailable metal in underground PM compared to ambient PM may be overestimated if only total PM metal concentration is considered.

4. Per mass of PM, underground PM is generally poor in organic carbon and secondary anions such as NO_3_^−^ and SO_4_^2−^, the latter potentially underlying the poor solubility of metals in underground PM.

5. As well as having a significantly increased PM mass concentration and PM metal concentration, underground railways also tend to have lower concentrations of traffic-related gaseous pollutants (e.g. NO_2_) compared to above-ground locations.

6. In vitro, underground PM appears to be better able to elicit ROS generation/antioxidant depletion than is ambient PM, likely related to underground PM transition metal composition.

7. Metal-related ROS generation appears at least partly to underlie the oxidative damage of DNA, and induction of antioxidant expression.

8. In vitro, inflammatory cytokine release in response to underground PM relative to ambient PM may depend on whether the cell type used is sensitive to endotoxin, which is generally less concentrated in underground PM compared to ambient PM. Thus, endotoxin-sensitive cells (e.g. macrophages), may appear to be especially sensitive to ambient PM, while endotoxin-insensitive cells (e.g. epithelial cells) may appear to be relatively less sensitive to ambient PM.

9. The heightened effects of underground PM compared to ambient PM, in terms of oxidative damage and effects where endotoxin is unlikely to be involved, are clearly able to outweigh the burden of toxicity which may be expected in urban PM as a result of increased PNC, compared to the lower PNC of an equal mass of underground PM.

10. The association of transition metals and ROS generation with various endpoints in vitro is not obviously consistently apparent in studies in vivo. This may be due to intrinsic differences between complex tissues and organs, comprising multiple interacting cell types, and simple cell cultures, or the use of PM concentrations in studies in vitro which are unlikely to be attained in normal in vivo environmental exposures.

11. On the basis of PM composition, underground PM may be associated with an increased risk of carcinogenicity and non-cancer health effects due to its metal-rich composition, which may outweigh the toxicity of PAH in ambient PM. This mainly relates to transition metals; studies have highlighted the potential risks associated with not only Fe, but also non-ferrous metals in underground PM, such as Cr, Ni, Co, Mn, and Cd, although these do not generally exceed exposure limits.

12. Controlled studies of short term exposure in underground stations have shown some associations of metals with respiratory endpoints, although these were not always associated with PM OP. However, the same studies have found other endpoints unrelated to PM metals, and instead related to components such as PM organic carbon and anions, both of which comprise relatively low proportions of underground PM mass compared to ambient PM.

13. Studies of underground railway workers, who are chronically exposed to air pollution in underground railway systems, have generally found little or no association of disease endpoints or exacerbations with working in the underground. However, these studies, of which there are only a small number, generally suffer from the use of relatively small sample sizes, while the working populations studied may represent a relatively non-susceptible subset of the population. Therefore, it is difficult to draw robust conclusions from them, especially in terms of how the commuting population may be affected.

14. The closest approximation to underground PM exposures elsewhere may be in steel mills, although these are generally lower in PM mass concentration and PM metal content than underground PM exposures, and also generally higher in pollutant gases (e.g. NO_2_, SO_2_) than underground. Notably, as for the underground, studies of workers in steel mills/steel plants have also found no consistent clinically significant effects.

15. The RAPTES studies and a study of the effects of a steel-type Fe-Mn signature in ambient PM in New York City have suggested that transition metals may be more associated than ambient PM with effects on lung function, but that cardiovascular endpoints seem to be driven by PM components in which underground PM is less rich. However, these studies per se do not provide sufficient evidence for a likely effect of underground PM exposure.

Overall, while both the increased PM mass concentration and metal content of underground railway PM are suggestive of, and seem to be responsible for, effects on various toxicological endpoints in vitro, there is much less evidence to indicate overt toxicity of underground PM exposure in vivo. While it appears that the unusual composition of underground PM may underlie some of its effects, and that some of the effects of underground PM may be different to the effects of ambient PM, it is certainly not the case that these effects of underground PM are necessarily of a greater magnitude than those of ambient PM. Furthermore, there are PM components such as PAHs, and gaseous pollutants such as NO_2_, which are generally found at higher concentrations in ambient air compared to underground air, and which are also associated with health effects.

From the small number of studies, there is little evidence that the physicochemical characteristics of underground PM translate to a significantly increased risk of adverse health effects in underground railway workers or commuters, although it is clear that further work in this area is required. From a mechanistic viewpoint, more attention needs to be paid to the non-ferrous components of underground PM, ROS- and non-ROS-related mechanisms of toxicity of underground PM, alternative endpoints of PM toxicity as they become identified by epidemiological and omics research, and the distinct effects (if any) of acute exposure vs. chronic, repeated exposure over a lifetime, however short the individual exposures may be. In terms of understanding the effects of exposure in vivo, there is a particular need for well-designed studies with study populations significantly larger than those hitherto used, and where possible using different population groups stratified according to factors such as age, sex, and underlying disease. Furthermore, beyond the established PM-associated diseases of cardiovascular disease, asthma, and lung cancer, and given the potential for relatively-insoluble PM to cross the air-blood barrier [111, 112], studies of the effects of chronic exposure to underground PM should also examine other more recent associations with PM exposure, including idiopathic pulmonary fibrosis [113, 114], type 2 diabetes [115, 116], Alzheimer’s disease [117], and decreased cognitive function [118].

## Abbreviations

8-OHdG: 8-hydroxy-2′-deoxyguanosine
BALF: bronchoalveolar lavage fluid
CYP: cytochrome p450
DCF: dichlorofluorescein
DEP: diesel exhaust particulate matter
DTT: dithiothreitol
EC: elemental carbon
F_E_NO: fraction of exhaled nitric oxide
FEV1: forced expiratory volume in 1 s
FVC: forced vital capacity
HRV: heart rate variability
OC: organic carbon
OP: oxidative potential PAH – polyaromatic hydrocarbon
PAI-1: plasminogen activator inhibitor-1
PEF: peak expiratory flow
PGE2: prostaglandin E2
PM: particulate matter
PNC: particle number concentration
ROS: reactive oxygen species
UFPM: ultrafine particulate matter

## References

[1] Derrible S, Kennedy C (2009). Network analysis of world subway systems using updated graph theory. Transp Res Record.

[2] Sahin UA, Onat B, Stakeeva B, Ceran T, Karim P (2012). PM10 concentrations and the size distribution of cu and Fe-containing particles in Istanbul's subway system. Transport Res D-Tr E.

[3] Querol X, Moreno T, Karanasiou A, Reche C, Alastuey A, Viana M (2012). Variability of levels and composition of PM10 and PM2.5 in the Barcelona metro system. Atmos Chem Phys.

[4] Jung HJ, Kim B, Ryu J, Maskey S, Kim JC, Sohn J (2010). Source identification of particulate matter collected at underground subway stations in Seoul, Korea using quantitative single-particle analysis. Atmos Environ.

[5] Colombi C, Angius S, Gianelle V, Lazzarini M (2013). Particulate matter concentrations, physical characteristics and elemental composition in the Milan underground transport system. Atmos Environ.

[6] Moreno T, Perez N, Reche C, Martins V, de Miguel E, Capdevila M (2014). Subway platform air quality: assessing the influences of tunnel ventilation, train piston effect and station design. Atmos Environ.

[7] Moreno T, Reche C, Minguillon MC, Capdevila M, de Miguel E, Querol X (2017). The effect of ventilation protocols on airborne particulate matter in subway systems. Sci Total Environ.

[8] Loxham M, Cooper MJ, Gerlofs-Nijland ME, Cassee FR, Davies DE, Palmer MR (2013). Physicochemical characterization of airborne particulate matter at a mainline underground railway station. Environ Sci Technol.

[9] Eom HJ, Jung HJ, Sobanska S, Chung SG, Son YS, Kim JC (2013). Iron speciation of airborne subway particles by the combined use of energy dispersive electron probe X-ray microanalysis and Raman microspectrometry. Anal Chem.

[10] Kang S, Hwang H, Park Y, Kim H, Ro CU (2008). Chemical compositions of subway particles in Seoul, Korea determined by a quantitative single particle analysis. Environ Sci Technol.

[11] Chillrud SN, Grass D, Ross JM, Coulibaly D, Slavkovich V, Epstein D (2005). Steel dust in the new York City subway system as a source of manganese, chromium, and iron exposures for transit workers. J Urban Health.

[12] Moreno T, Martins V, Querol X, Jones T, BéruBé K, Minguillón MC (2015). A new look at inhalable metalliferous airborne particles on rail subway platforms. Sci Total Environ.

[13] Xu B, Hao JL (2017). Air quality inside subway metro indoor environment worldwide: a review. Environ Int.

[14] Nieuwenhuijsen MJ, Gomez-Perales JE, Colvile RN (2007). Levels of particulate air pollution, its elemental composition, determinants and health effects in metro systems. Atmos Environ.

[15] Klepczynska Nystrom A, Svartengren M, Grunewald J, Pousette C, Rodin I, Lundin A (2010). Health effects of a subway environment in healthy volunteers. Eur Respir J.

[16] Vignali DAA, Collison LW, Workman CJ (2008). How regulatory T cells work. Nat Rev Immunol.

[17] Akdis M, Aab A, Altunbulakli C, Azkur K, Costa RA, Crameri R (2016). Interleukins (from IL-1 to IL-38), interferons, transforming growth factor beta, and TNF-alpha: receptors, functions, and roles in diseases. J Allergy Clin Immunol.

[18] Deiuliis JA, Kampfrath T, Zhong JX, Oghumu S, Maiseyeu A, Chen LC (2012). Pulmonary T cell activation in response to chronic particulate air pollution. Am J Phys Lung Cell Mol Phys.

[19] Nadeau K, McDonald-Hyman C, Noth EM, Pratt B, Hammond SK, Balmes J (2010). Ambient air pollution impairs regulatory T-cell function in asthma. J Allergy Clin Immunol.

[20] Bigert C, Alderling M, Svartengren M, Plato N, Gustavsson P (2011). No short-term respiratory effects among particle-exposed employees in the Stockholm subway. Scand J Work Environ Health.

[21] Klepczynska-Nystrom A, Larsson BM, Grunewald J, Pousette C, Lundin A, Eklund A (2012). Health effects of a subway environment in mild asthmatic volunteers. Respir Med.

[22] Doolittle RF (2017). The conversion of fibrinogen to fibrin: A brief history of some key events. Matrix Biol.

[23] Danesh J, Lewington S, Thompson SG, Lowe GDO, Collins R, Collaboration FS (2005). Plasma fibrinogen level and the risk of major cardiovascular diseases and nonvascular mortality - an individual participant meta-analysis. Jama-J Am Med Assoc.

[24] Erickson LA, Ginsberg MH, Loskutoff DJ (1984). Detection and partial characterization of an inhibitor of plasminogen activator in human platelets. J Clin Invest.

[25] Brook RD, Rajagopalan S, Pope CA, Brook JR, Bhatnagar A, Diez-Roux AV (2010). Particulate matter air pollution and cardiovascular disease: An update to the scientific statement from the American Heart Association. Circulation.

[26] Ljungman P, Bellander T, Schneider A, Breitner S, Forastiere F, Hampel R (2009). Modification of the interleukin-6 response to air pollution by interleukin-6 and fibrinogen polymorphisms. Environ Health Perspect.

[27] Bigert C, Alderling M, Svartengren M, Plato N, de Faire U, Gustavsson P (2008). Blood markers of inflammation and coagulation and exposure to airborne particles in employees in the Stockholm underground. Occup Environ Med.

[28] Lundstrom SL, Levanen B, Nording M, Klepczynska-Nystrom A, Skold M, Haeggstrom JZ (2011). Asthmatics exhibit altered oxylipin profiles compared to healthy individuals after subway air exposure. PLoS One.

[29] Gouveia-Figueira S, Karimpour M, Bosson JA, Blomberg A, Unosson J, Pourazar J (2017). Mass spectrometry profiling of oxylipins, endocannabinoids, and N-acylethanolamines in human lung lavage fluids reveals responsiveness of prostaglandin E2 and associated lipid metabolites to biodiesel exhaust exposure. Anal Bioanal Chem.

[30] Liu WT, Ma CM, Liu IJ, Han BC, Chuang HC, Chuang KJ (2015). Effects of commuting mode on air pollution exposure and cardiovascular health among young adults in Taipei, Taiwan. Int J Hyg Environ Health.

[31] Thayer JF, Yamamoto SS, Brosschot JF (2010). The relationship of autonomic imbalance, heart rate variability and cardiovascular disease risk factors. Int J Cardiol.

[32] Kelly FJ, Fussell JC (2017). Role of oxidative stress in cardiovascular disease outcomes following exposure to ambient air pollution. Free Radic Biol Med.

[33] Bigert C, Klerdal K, Hammar N, Gustavsson P (2007). Myocardial infarction in Swedish subway drivers. Scand J Work Environ Health.

[34] Gustavsson P, Bigert C, Pollan M (2008). Incidence of lung cancer among subway drivers in Stockholm. Am J Ind Med.

[35] Grass DS, Ross JM, Family F, Barbour J, Simpson HJ, Coulibaly D (2010). Airborne particulate metals in the new York City subway: a pilot study to assess the potential for health impacts. Environ Res.

[36] Mehrdad R, Aghdaei S, Pouryaghoub G (2015). Urinary 8-hydroxy-deoxyguanosine as a biomarker of oxidative DNA damage in employees of subway system. Acta Med Iran.

[37] Loxham M (2015). Harmful effects of particulate air pollution: identifying the culprits. Respirology.

[38] Seaton A, Cherrie J, Dennekamp M, Donaldson K, Hurley JF, Tran CL (2005). The London underground: dust and hazards to health. Occup Environ Med.

[39] Peters A, Wichmann HE, Tuch T, Heinrich J, Heyder J (1997). Respiratory effects are associated with the number of ultrafine particles. Am J Respir Crit Care Med.

[40] Karlsson HL, Nilsson L, Moller L (2005). Subway particles are more genotoxic than street particles and induce oxidative stress in cultured human lung cells. Chem Res Toxicol.

[41] Karlsson HL, Ljungman AG, Lindbom J, Moller L (2006). Comparison of genotoxic and inflammatory effects of particles generated by wood combustion, a road simulator and collected from street and subway. Toxicol Lett.

[42] Karlsson HL, Holgersson A, Moller L (2008). Mechanisms related to the genotoxicity of particles in the subway and from other sources. Chem Res Toxicol.

[43] van Klaveren RJ, Nemery B (1999). Role of reactive oxygen species in occupational and environmental obstructive pulmonary diseases. Curr Opin Pulm Med.

[44] Vidrio E, Jung H, Anastasio C (2008). Generation of hydroxyl radicals from dissolved transition metals in surrogate lung fluid solutions. Atmos Environ.

[45] Mudway IS, Stenfors N, Duggan ST, Roxborough H, Zielinski H, Marklund SL (2004). An in vitro and in vivo investigation of the effects of diesel exhaust on human airway lining fluid antioxidants. Arch Biochem Biophys.

[46] van der Vliet A, O'Neill CA, Cross CE, Koostra JM, Volz WG, Halliwell B (1999). Determination of low-molecular-mass antioxidant concentrations in human respiratory tract lining fluids. Am J Phys.

[47] Lu SL, Liu DY, Zhang WC, Liu PW, Fei Y, Gu Y (2015). Physico-chemical characterization of PM2.5 in the microenvironment of Shanghai subway. Atmos Res.

[48] Salma I, Posfai M, Kovacs K, Kuzmann E, Homonnay Z, Posta J (2009). Properties and sources of individual particles and some chemical species in the aerosol of a metropolitan underground railway station. Atmos Environ.

[49] Jung HJ, Kim B, Malek MA, Koo YS, Jung JN, Son YS (2012). Chemical speciation of size-segregated floor dusts and airborne magnetic particles collected at underground subway stations in Seoul, Korea. J Hazard Mater.

[50] Pugin J, Schurermaly CC, Leturcq D, Moriarty A, Ulevitch RJ, Tobias PS (1993). Lipopolysaccharide activation of human endothelial and epithelial-cells is mediated by lipopolysaccharide-binding protein and soluble Cd14. Proc Natl Acad Sci U S A.

[51] Shen HY, Anastasio C (2012). A comparison of hydroxyl radical and hydrogen peroxide generation in ambient particle extracts and laboratory metal solutions. Atmos Environ.

[52] Charrier JG, Anastasio C (2011). Impacts of antioxidants on hydroxyl radical production from individual and mixed transition metals in a surrogate lung fluid. Atmos Environ.

[53] Brown DM, Kinloch IA, Bangert U, Windle AH, Walter DM, Walker GS (2007). An in vitro study of the potential of carbon nanotubes and nanofibres to induce inflammatory mediators and frustrated phagocytosis. Carbon.

[54] Poland CA, Duffin R, Kinloch I, Maynard A, Wallace WAH, Seaton A (2008). Carbon nanotubes introduced into the abdominal cavity of mice show asbestos-like pathogenicity in a pilot study. Nat Nanotechnol.

[55] Lindbom J, Gustafsson M, Blomqvist G, Dahl A, Gudmundsson A, Swietlicki E (2006). Exposure to wear particles generated from studded tires and pavement induces inflammatory cytokine release from human macrophages. Chem Res Toxicol.

[56] Lindbom J, Gustafsson M, Blomqvist G, Dahl A, Gudmundsson A, Swietlicki E (2007). Wear particles generated from studded tires and pavement induces inflammatory reactions in mouse macrophage cells. Chem Res Toxicol.

[57] Bachoual R, Boczkowski J, Goven D, Amara N, Tabet L, On D (2007). Biological effects of particles from the Paris subway system. Chem Res Toxicol.

[58] Jung MH, Kim HR, Park YJ, Park DS, Chung KH, Oh SM (2012). Genotoxic effects and oxidative stress induced by organic extracts of particulate matter (PM10) collected from a subway tunnel in Seoul, Korea. Mutat Res-Gen Tox En.

[59] Tompkins LM, Wallace AD (2007). Mechanisms of cytochrome P450 induction. J Biochem Mol Toxicol.

[60] Loxham M, Morgan-Walsh RJ, Cooper MJ, Blume C, Swindle EJ, Dennison PW (2015). The effects on bronchial epithelial mucociliary cultures of coarse, fine, and ultrafine particulate matter from an underground railway station. Toxicol Sci.

[61] Blume C, Davies DE (2013). In vitro and ex vivo models of human asthma. Eur J Pharm Biopharm.

[62] Spagnolo AM, Ottria G, Perdelli F, Cristina ML (2015). Chemical characterisation of the coarse and fine particulate matter in the environment of an underground railway system: cytotoxic effects and oxidative stress-a preliminary study. Int J Environ Res Public Health.

[63] Zielinski H, Mudway IS, Berube KA, Murphy S, Richards R, Kelly FJ (1999). Modeling the interactions of particulates with epithelial lining fluid antioxidants. Am J Phys Lung Cell Mol Phys.

[64] Borm PJA, Kelly F, Kunzli N, Schins RPF, Donaldson K (2007). Oxidant generation by particulate matter: from biologically effective dose to a promising, novel metric. Occup Environ Med.

[65] Ayres JG, Borm P, Cassee FR, Castranova V, Donaldson K, Ghio A (2008). Evaluating the toxicity of airborne particulate matter and nanoparticles by measuring oxidative stress potential - a workshop report and consensus statement. Inhal Toxicol.

[66] Moreno T, Kelly FJ, Dunster C, Oliete A, Martins V, Reche C (2017). Oxidative potential of subway PM2.5. Atmos Environ.

[67] Janssen NA, Yang A, Strak M, Steenhof M, Hellack B, Gerlofs-Nijland ME (2014). Oxidative potential of particulate matter collected at sites with different source characteristics. Sci Total Environ.

[68] Kim KH, Ho DX, Jeon JS, Kim JC (2012). A noticeable shift in particulate matter levels after platform screen door installation in a Korean subway station. Atmos Environ.

[69] Gali NK, Jiang SY, Yang FH, Sun L, Ning Z (2017). Redox characteristics of size-segregated PM from different public transport microenvironments in Hong Kong. Air Qual Atmos Hlth.

[70] Gali NK, Yang FH, Jiang SY, Chan KL, Sun L, Ho KF (2015). Spatial and seasonal heterogeneity of atmospheric particles induced reactive oxygen species in urban areas and the role of water-soluble metals. Environ Pollut.

[71] Stone V, Miller MR, Clift MJD, Elder A, Mills NL, Moller P (2017). Nanomaterials versus ambient ultrafine particles: an opportunity to exchange toxicology knowledge. Environ Health Perspect.

[72] Burnett RT, Pope CA, Ezzati M, Olives C, Lim SS, Mehta S (2014). An integrated risk function for estimating the global burden of disease attributable to ambient fine particulate matter exposure. Environ Health Perspect.

[73] Park DU, Ha KC (2008). Characteristics of PM10, PM2.5, CO2 and CO monitored in interiors and platforms of subway train in Seoul, Korea. Environ Int.

[74] Murruni LG, Solanes V, Debray M, Kreiner AJ, Davidson J, Davidson M (2009). Concentrations and elemental composition of particulate matter in the Buenos Aires underground system. Atmos Environ.

[75] Kelly FK, Mudway J, Blomberg A, Frew A, Sandstrom T (1999). Altered lung antioxidant status in patients with mild asthma. Lancet..

[76] Bucchieri F, Puddicombe SM, Lordan JL, Richter A, Buchanan D, Wilson SJ (2002). Asthmatic bronchial epithelium is more susceptible to oxidant-induced apoptosis. Am J Respir Cell Mol Biol.

[77] Strak M, Steenhof M, Godri K, Gosens I, Cassee F, Hoek G (2011). Physical, chemical, and oxidative characterization of particles from locations with contrast in local source emissions: exposure and health assessment in the RAPTES study. Epidemiology.

[78] Steenhof M, Gosens I, Strak M, Godri KJ, Hoek G, Cassee FR (2011). In vitro toxicity of particulate matter (PM) collected at different sites in the Netherlands is associated with PM composition, size fraction and oxidative potential - the RAPTES project. Part Fibre Toxicol.

[79] Dweik RA, Boggs PB, Erzurum SC, Irvin CG, Leigh MW, Lundberg JO (2011). An official ATS clinical practice guideline: interpretation of exhaled nitric oxide levels (FENO) for clinical applications. Am J Respir Crit Care Med.

[80] Strak M, Janssen NA, Godri KJ, Gosens I, Mudway IS, Cassee FR (2012). Respiratory health effects of airborne particulate matter: the role of particle size, composition, and oxidative potential-the RAPTES project. Environ Health Perspect.

[81] Steenhof M, Mudway IS, Gosens I, Hoek G, Godri KJ, Kelly FJ (2013). Acute nasal pro-inflammatory response to air pollution depends on characteristics other than particle mass concentration or oxidative potential: the RAPTES project. Occup Environ Med.

[82] Strak M, Hoek G, Godri KJ, Gosens I, Mudway IS, van Oerle R (2013). Composition of PM affects acute vascular inflammatory and coagulative markers - the RAPTES project. PLoS One.

[83] Strak M, Hoek G, Steenhof M, Kilinc E, Godri KJ, Gosens I (2013). Components of ambient air pollution affect thrombin generation in healthy humans: the RAPTES project. Occup Environ Med.

[84] Steenhof M, Janssen NA, Strak M, Hoek G, Gosens I, Mudway IS (2014). Air pollution exposure affects circulating white blood cell counts in healthy subjects: the role of particle composition, oxidative potential and gaseous pollutants - the RAPTES project. Inhal Toxicol.

[85] Kam W, Ning Z, Shafer MM, Schauer JJ, Sioutas C (2011). Chemical characterization and redox potential of coarse and fine particulate matter (PM) in underground and ground-level rail systems of the Los Angeles metro. Environ Sci Technol..

[86] Kam W, Delfino RJ, Schauer JJ, Sioutas C (2013). A comparative assessment of PM2.5 exposures in light-rail, subway, freeway, and surface street environments in Los Angeles and estimated lung cancer risk. Environ Sci-Proc Imp.

[87] Lovett C, Shirmohammadi F, Sowlat MH, Sioutas C (2018). Commuting in Los Angeles: cancer and non-cancer health risks of roadway, light-rail and subway transit routes. Aerosol Air Qual Res.

[88] Keith LH (2015). The source of US EPA's sixteen PAH priority pollutants. Polycycl Aromat Comp.

[89] Costa M (1997). Toxicity and carcinogenicity of Cr(VI) in animal models and humans. Crit Rev Toxicol.

[90] Cohen MD, Kargacin B, Klein CB, Costa M (1993). Mechanisms of chromium carcinogenicity and toxicity. Crit Rev Toxicol.

[91] Chillrud SN, Epstein D, Ross JM, Sax SN, Pederson D, Spengler JD (2004). Elevated airborne exposures of teenagers to manganese, chromium, and iron from steel dust and new York City's subway system. Environ Sci Technol.

[92] Cao SJ, Kong XR, Li LY, Zhang WR, Ye ZP, Deng YL (2017). An investigation of the PM2.5 and NO2 concentrations and their human health impacts in the metro subway system of Suzhou, China. Environ Sci-Proc Imp.

[93] Hurley JF, Cherrie J, Donaldson K, Seaton A, Tran CL (2003). Assessment of health effects of long-term occupational exposure to tunnel dust in the London underground. Vol. TM/03/02.

[94] Murphy AB (2010). The effects of metal vapour in arc welding. J Phys D Appl Phys.

[95] Cakmak S, Dales R, Kauri LM, Mahmud M, Van Ryswyk K, Vanos J (2014). Metal composition of fine particulate air pollution and acute changes in cardiorespiratory physiology. Environ Pollut.

[96] Dales R, Kauri LM, Cakmak S, Mahmud M, Weichenthal SA, Van Ryswyk K (2013). Acute changes in lung function associated with proximity to a steel plant: a randomized study. Environ Int.

[97] Shutt RH, Kauri LM, Weichenthal S, Kumarathasan P, Vincent R, Thomson EM (2017). Exposure to air pollution near a steel plant is associated with reduced heart rate variability: a randomised crossover study. Environ Health.

[98] Pelletier G, Rigden M, Kauri LM, Shutt R, Mahmud M, Cakmak S (2017). Associations between urinary biomarkers of oxidative stress and air pollutants observed in a randomized crossover exposure to steel mill emissions. Int J Hyg Environ Health.

[99] Pavanello S, Bonzini M, Angelici L, Motta V, Pergoli L, Hoxha M (2016). Extracellular vesicle-driven information mediates the long-term effects of particulate matter exposure on coagulation and inflammation pathways. Toxicol Lett.

[100] Yu SL, Lee DC, Sohn HA, Lee SY, Jeon HS, Lee JH (2016). Homeobox A9 directly targeted by miR-196b regulates aggressiveness through nuclear factor-kappa B activity in non-small cell lung cancer cells. Mol Carcinog.

[101] Panda AC, Sahu I, Kulkarni SD, Martindale JL, Abdelmohsen K, Vindu A (2014). miR-196b-mediated translation regulation of mouse insulin2 via the 5'UTR. PLoS One.

[102] Bollati V, Angelici L, Rizzo G, Pergoli L, Rota F, Hoxha M (2015). Microvesicle-associated microRNA expression is altered upon particulate matter exposure in healthy workers and in A549 cells. J Appl Toxicol.

[103] Tarantini L, Bonzini M, Tripodi A, Angelici L, Nordio F, Cantone L (2013). Blood hypomethylation of inflammatory genes mediates the effects of metal-rich airborne pollutants on blood coagulation. Occup Environ Med.

[104] Cantone L, Nordio F, Hou L, Apostoli P, Bonzini M, Tarantini L (2011). Inhalable metal-rich air particles and histone H3K4 dimethylation and H3K9 acetylation in a cross-sectional study of steel workers. Environ Health Perspect.

[105] Pope CA (1989). Respiratory disease associated with community air pollution and a steel mill, Utah Valley. Am J Public Health.

[106] Dye JA, Lehmann JR, McGee JK, Winsett DW, Ledbetter AD, Everitt JI (2001). Acute pulmonary toxicity of particulate matter filter extracts in rats: coherence with epidemiologic studies in Utah Valley residents. Environ Health Perspect.

[107] Molinelli AR, Madden MC, McGee JK, Stonehuerner JG, Ghio AJ (2002). Effect of metal removal on the toxicity of airborne particulate matter from the Utah Valley. Inhal Toxicol.

[108] Pagan I, Costa DL, McGee JK, Richards JH, Dye JA, Dykstra MJ (2003). Metals mimic airway epithelial injury induced by in vitro exposure to Utah Valley ambient particulate matter extracts. J Toxicol Env Heal A.

[109] Lall R, Ito K, Thurston GD (2011). Distributed lag analyses of daily hospital admissions and source-apportioned fine particle air pollution. Environ Health Perspect.

[110] Lippmann M (2014). Toxicological and epidemiological studies of cardiovascular effects of ambient air fine particulate matter (PM2.5) and its chemical components: coherence and public health implications. Crit Rev Toxicol.

[111] Maher BA, Ahmed IA, Karloukovski V, MacLaren DA, Foulds PG, Allsop D (2016). Magnetite pollution nanoparticles in the human brain. Proc Natl Acad Sci U S A.

[112] Miller MR, Raftis JB, Langrish JP, McLean SG, Samutrtai P, Connell SP (2017). Inhaled nanoparticles accumulate at sites of vascular disease. ACS Nano.

[113] Winterbottom CJ, Shah RJ, Patterson KC, Kreider ME, Panettieri RA, Rivera-Lebron B (2018). Exposure to ambient particulate matter is associated with accelerated functional decline in idiopathic pulmonary fibrosis. Chest.

[114] Taskar VS, Coultas DB (2006). Is idiopathic pulmonary fibrosis an environmental disease?. Proc Am Thorac Soc.

[115] Eze IC, Hemkens LG, Bucher HC, Hoffmann B, Schindler C, Kunzli N (2015). Association between ambient air pollution and diabetes mellitus in Europe and North America: systematic review and meta-analysis. Environ Health Perspect.

[116] Strak M, Janssen N, Beelen R, Schmitz O, Vaartjes I, Karssenberg D (2017). Long-term exposure to particulate matter, NO2 and the oxidative potential of particulates and diabetes prevalence in a large national health survey. Environ Int.

[117] Jung CR, Lin YT, Hwang BF (2015). Ozone, particulate matter, and newly diagnosed Alzheimer's disease: a population-based cohort study in Taiwan. J Alzheimers Dis.

[118] Zhang X, Chen X, Zhang XB (2018). The impact of exposure to air pollution on cognitive performance. Proc Natl Acad Sci U S A.

